# Which factors influence the extent of indoor transmission of SARS-CoV-2? A rapid evidence review

**DOI:** 10.7189/jogh.11.10002

**Published:** 2021-04-03

**Authors:** Lara Goodwin, Toneka Hayward, Prerna Krishan, Gemma Nolan, Madhurima Nundy, Kayla Ostrishko, Antonio Attili, Salva Barranco Cárceles, Emmanuel I Epelle, Roman Gabl, Evanthia J Pappa, Mateusz Stajuda, Simone Zen, Marshall Dozier, Niall Anderson, Ignazio M Viola, Ruth McQuillan

**Affiliations:** 1 Usher Institute, University of Edinburgh, Edinburgh, UK; 2 School of Engineering, University of Edinburgh, Edinburgh, UK; 3 Information Services, University of Edinburgh, Edinburgh, UK

## Abstract

**Background:**

This rapid evidence review identifies and integrates evidence from epidemiology, microbiology and fluid dynamics on the transmission of SARS-CoV-2 in indoor environments.

**Methods:**

Searches were conducted in May 2020 in PubMed, medRxiv, arXiv, Scopus, WHO COVID-19 database, Compendex & Inspec. We included studies reporting data on any indoor setting except schools, any indoor activities and any potential means of transmission. Articles were screened by a single reviewer, with rejections assessed by a second reviewer. We used Joanna Briggs Institute and Critical Appraisal Skills Programme tools for evaluating epidemiological studies and developed bespoke tools for the evaluation of study types not covered by these instruments. Data extraction and quality assessment were conducted by a single reviewer. We conducted a meta-analysis of secondary attack rates in household transmission. Otherwise, data were synthesised narratively.

**Results:**

We identified 1573 unique articles. After screening and quality assessment, fifty-eight articles were retained for analysis. Experimental evidence from fluid mechanics and microbiological studies demonstrates that aerosolised transmission is theoretically possible; however, we found no conclusive epidemiological evidence of this occurring. The evidence suggests that ventilation systems have the potential to decrease virus transmission near the source through dilution but to increase transmission further away from the source through dispersal. We found no evidence for faecal-oral transmission. Laboratory studies suggest that the virus survives for longer on smooth surfaces and at lower temperatures. Environmental sampling studies have recovered small amounts of viral RNA from a wide range of frequently touched objects and surfaces; however, epidemiological studies are inconclusive on the extent of fomite transmission. We found many examples of transmission in settings characterised by close and prolonged indoor contact. We estimate a pooled secondary attack rate within households of 11% (95% confidence interval (CI) = 9, 13). There were insufficient data to evaluate the transmission risks associated with specific activities. Workplace challenges related to poverty warrant further investigation as potential risk factors for workplace transmission. Fluid mechanics evidence on the physical properties of droplets generated by coughing, speaking and breathing reinforce the importance of maintaining 2 m social distance to reduce droplet transmission.

**Conclusions:**

This review provides a snap-shot of evidence on the transmission of SARS-CoV-2 in indoor environments from the early months of the pandemic. The overall quality of the evidence was low. As the quality and quantity of available evidence grows, it will be possible to reach firmer conclusions on the risk factors for and mechanisms of indoor transmission.

It is well established that SARS-CoV-2 is readily transmitted in indoor environments; however, questions remain about the relative importance of different transmission mechanisms, the risks associated with non-clinical indoor environments and activities and the role of ventilation and plumbing systems in mitigating or amplifying transmission. Although other reviews address aspects of these questions [1-3] there are no published reviews which integrate evidence from different disciplines in order to address questions of direct and immediate relevance to decision-makers. This rapid evidence review identifies and integrates evidence from three disciplines, each of which has distinct strengths and limitations. Descriptive epidemiological studies can identify likely routes of transmission; however, such observational findings have a high risk of bias and rarely provide sufficiently detailed data to establish transmission mechanisms with certainty. The discipline of fluid mechanics provides important insights into the physical behaviour of small and large droplets under different environmental conditions and about the size and velocity profiles of particles emitted during speech, breathing, coughing and sneezing. However, numerical modelling studies and experiments conducted under strictly controlled laboratory conditions do not account for all aspects of physical reality, and are not concerned with the viability or infectivity of virus particles. Microbiological experiments investigate the viability of the virus under different environmental and time periods under controlled laboratory conditions; however again, the results may not be generalizable to the real world.

The purpose of this review is to integrate evidence from epidemiological, microbiological and fluid mechanics studies on the transmission of SARS-CoV-2 in indoor, non-clinical settings in order to answer ten specific questions:

1. What evidence is there for aerosolised transmission?
2. What evidence is there for faecal-oral transmission?
3. What evidence is there regarding the role of ventilation systems in indoor transmission?
4. What evidence is there regarding the role of plumbing systems in indoor transmission?
5. What evidence is there regarding transmission via different indoor surfaces (materials and specific objects)?
6. What evidence is there for the transmission in indoor residential settings?
7. What evidence is there for transmission in indoor workplace settings?
8. What evidence is there for transmission in other indoor settings (social, community, leisure, religious, public transport)?
9. Do particular activities convey greater risk (e.g. shouting, singing, eating together, sharing bedrooms)?
10. What evidence is there for the appropriate length of distancing between people?

## METHODS

### Search strategy

We designed two separate search strategies: one to identify epidemiological and microbiological papers and the other focused on fluid mechanics papers (mechanistic studies). We searched PubMed, medRxiv, arXiv, Scopus, WHO COVID-19 database, Compendex & Inspec. Searches were collaboratively developed by two reviewers (MD and LG) and results exported on 20 and 21 May 2020. Full search details are in Appendix S1 in the **Online Supplementary Document**. The search strategy and screening, data extraction and quality assessment procedures are summarised in **Table 1**.

**Table 1:** Summary of search, screening and quality assessment strategies

Discipline	Epidemiology	Microbiology	Mechanics of indoor transmission
Summary of search strategy (see Appendix S1 in the **Online Supplementary Document** for full search strategy)	SARS-CoV-2 AND transmission AND indoor	SARS-CoV-2 AND transmission AND indoor	SARS-CoV-2 AND transmission AND mechanistic terms
Databases searched	PubMed, medRxiv	PubMed, medRxiv	PubMed, medRxiv, arXiv, Scopus, WHO COVID-19 database, Compendex & Inspec
Screening criteria	Inclusion: SARS-CoV-2; any indoor settings except schools and clinical settings; any indoor activities; any potential means of transmission. Exclusion: other respiratory viruses; schools; clinical settings; studies focused on clinical characteristics of cases; non-descriptive (statistical modelling) studies aiming to predict future events.	Inclusion: SARS-CoV-2; analysis of swabs collected from any indoor settings, including clinical, except ITU/operating theatres, where aerosol-generating procedures routinely carried out; any potential means of transmission; laboratory studies under controlled conditions. Exclusion: other respiratory viruses; clinical settings such as ITU and operating theatres, where aerosol-generating procedures routinely carried out	Inclusion: any respiratory virus; any indoor setting except clinical setting where aerosol-generating procedures routinely carried out; any mechanism with potential to influence indoor transmission (eg, air conditioning, ventilation, plumbing); numerical simulation studies modelling fluid mechanics. Exclusion: clinical settings such as ITU and operating theatres, where aerosol-generating procedures routinely carried out
Quality assessment	Cross-sectional, case series and case reports – JBI checklists [4]. Other epidemiological studies – CASP checklists [4]. Contact tracing/cluster analysis studies – checklist adapted from JBI case series checklist.	Bespoke checklist [5-7]	Expert critical appraisal [8-10]

### Inclusion and exclusion criteria

We included studies reporting data on any indoor setting except schools, which is addressed elsewhere in a living systematic review [11], any indoor activities and any potential means of transmission. Other screening criteria differed according to study discipline, as follows.

Epidemiological studies: We excluded studies of transmission within clinical settings and studies focusing purely on the clinical characteristics of cases. We also excluded statistical modelling studies aiming to predict future outcomes, as opposed to descriptive studies characterising past events.

Microbiological studies: We included studies involving the testing of swabs taken from “real world” settings for the presence of SARS-CoV-2. As most of these were conducted in hospital settings, we included studies from both clinical and non-clinical settings. However, to maximise the transferability and generalisability of these findings to non-clinical indoor settings, we excluded microbiological studies of samples collected in areas of the hospital such as operating theatres and Intensive Care Units (ICU) where aerosol-generating procedures are routinely carried out. We also included laboratory studies investigating the persistence and viability of the virus under different controlled conditions.

Mechanistic studies: We included articles reporting data on any respiratory virus, numerical simulation studies focusing on the mechanisms of transmission and studies investigating any mechanisms with potential to influence transmission in indoor environments, such as ventilation, air conditioning or plumbing systems.

### Screening procedures

Articles were screened by two separate teams (LG, GN, PK, TH, RN, KO for epidemiological and microbiological studies and AA, SBC, EIE, RG, EP, MS, IMV, SZ for mechanistic studies). Title and abstract and full text screening were conducted by one reviewer within each team, with rejections assessed by a second reviewer.

### Data extraction and quality assessment

Data extraction and quality assessment for each article was conducted by a single reviewer, as above. A range of critical appraisal tools was employed, according to study design: case series and case reports were evaluated using Joanna Briggs Institute checklists [4]. We adapted a quality assessment tool for epidemiological outbreak cluster studies from the Joanna Briggs Institute checklist for critically appraising case series [4]. We adapted existing tools for the quality appraisal of laboratory experimental studies [5-7]. Details of adapted tools are provided in Appendix S2 in the **Online Supplementary Document**. For other epidemiological study designs, we used Critical Appraisal Skills Programme (CASP) checklists [12]. The overall quality of the epidemiological evidence on each research question was assessed by a single reviewer (RM) on the basis of the GRADE system [13]. Observational epidemiological studies were assigned an a priori grading of low, which could be downgraded on the basis of critical appraisal or upgraded on the basis of consistency across different studies and study designs. Mechanistic and numerical simulation studies were appraised by an expert in the field (IMV, SBC, EP, SZ, MS), based on three sources [8-10] (Appendix S2 in the **Online Supplementary Document**). Data extraction was limited to a minimal set of required data items: study question addressed by the article, study design and summary of methods, indoor context, outcome measure, relevant results.

### Data synthesis

Data heterogeneity was such that results were synthesised narratively, except for the results on secondary attack rates within households, which were meta-analysed using a fixed effect model in R 3.6.3 [14] using the *rma.uni()* function in the metafor package [15]. I^2^ and Cochrane’s Q were calculated to assess heterogeneity. For consistency, the same function was used to estimate confidence intervals for SAR in individual studies that were not included in pooled estimates. A fixed effects analysis was chosen because the number of studies was relatively small, and thus a simpler underlying model (fewer assumptions/parameters required) was likely to be better estimated: in addition, there is reasonably good theoretical and simulation evidence that fixed effects models are relatively robust to moderate heterogeneity [16]. There was little evidence of heterogeneity in the data but the number of studies was too small for such evidence to accumulate.

## RESULTS

**Figure 1:**
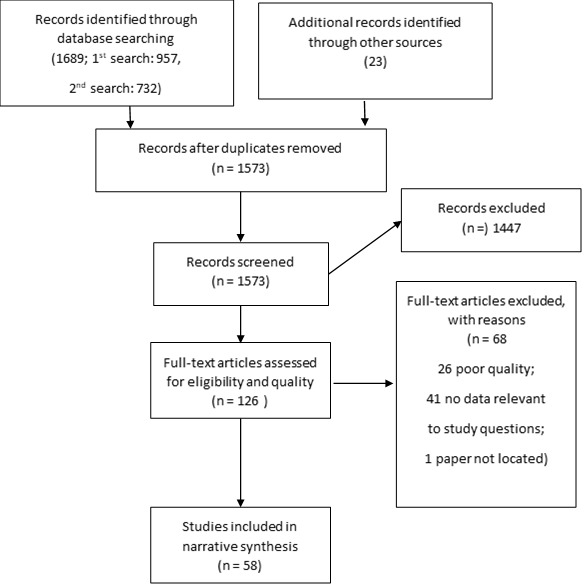
PRISMA diagram.

After the removal of duplicates, a total of 1573 articles were identified. A total of 1447 were rejected through title and abstract screening and a further 68 were rejected at the full-text screening stage and quality assessment stage. Forty-one did not provide data relevant to study questions, 26 were poor quality and one article could not be retrieved. Fifty-eight articles were retained for analysis (Appendix S3 in the **Online Supplementary Document**). This information is summarised in the PRISMA diagram (**Figure 1**). We report the results on each of the review questions separately, integrating the epidemiological, microbiological and fluid mechanics evidence.

### What evidence is there for aerosolised transmission?

**Table 2** summarises the evidence we identified on aerosolised transmission. The discipline of fluid mechanics provides important insights into the physical behaviour of respiratory droplets in the air. Respiratory droplets range in size from <10 μm to >1000 μm. Larger droplets follow a ballistic trajectory, falling to the ground within a few metres, the exact distance depending on the force with which they are ejected [17,18,36-38]. Smaller droplets (diameters of the order of 10 μm or smaller) fall so slowly through the air that they have time to evaporate [38]. These very light, desiccated particles, or aerosols, can then remain suspended in the air, potentially for several hours [18,19], and can travel long distances on air flows before eventually landing [17,19,20]. Studies conducted following the 2003 SARS outbreak provided evidence consistent with aerosolised transmission within buildings, influenced by the effects of ventilation and plumbing systems [21-25]. In order to ascertain whether aerosolised transmission of SARS-CoV-2 is possible, it is first necessary to establish whether and for how long it is able to persist in the air. In a laboratory-based study, van Doremalen et al found that aerosolised particles of SARS-CoV-2 remained viable for 3 hours (median half-life 1.09 hours, 95% credible interval 0.64, 2.64) [26]. Taken together, the fluid mechanics and microbiological studies demonstrate that aerosolised transmission of SARS-CoV-2 is theoretically possible.

**Table 2:** Evidence relating to aerosolised transmission

Reference	Study type	Overall study quality from critical appraisal	Relevant results
Can aerosolised particles suspended in the air be transported around indoor environments? There is moderate to high quality evidence from experimental and numerical simulation fluid mechanics studies that aerosolised particles suspended in the air are readily transported around indoor environments.			
[17]	Experimental fluid mechanics study - physical properties and behaviour of different particle sizes	High – very confident that the estimated effect is close to the true effect	Larger droplets (diameters of the order of 100-1000 μm) follow a ballistic trajectory (ie, they fall mostly under the influence of gravity) and reach the ground within a few seconds and without time to evaporate. The distance they travel before landing depends on (among other factors) how they were generated: those generated by coughing travel about 2 m before falling to the ground. Small droplets (aerosols) behave differently. Because they are small, they fall so slowly through the air that they have time to evaporate and can then remain suspended in the air for long periods. Aerosolised particles are ejected in a jet-like flux which, within a few metres, increases in diameter from a few centimetres to tens of centimetres. This flux bends upwards because it is warmer than the surrounding air. These particles can thus travel long distances on air flows before eventually landing.
[18]	Non-systematic review on the flow physics of COVID-19	Low – the estimated effect may be substantially different to the true effect	Desiccated particles (aerosols) can remain suspended in the air potentially indefinitely.
[19]	Experimental fluid dynamics study investigating the pattern of air flow within a commercial aircraft.	High – very confident that the estimated effect is close to the true effect	Illustration of the potential risk posed by aerosols – experiment showing that aerosols emitted above mid-body height would tend to remain at vertical elevations corresponding to the breathing levels of seated passengers in an aircraft carriage
[20]	Numerical simulation study investigating air flow patterns in a high speed train carriage.	Moderate – the estimated effect is likely to be close to the true effect but there is a possibility that it is substantially different	Numerical simulation study showing that aerosol dispersal is possible within a train coach
Is there evidence for aerosolised transmission of other human coronaviruses (SARS)? There is moderate to high quality evidence from fluid mechanics studies and low quality observational epidemiological evidence that aerosolised transmission played a role in the SARS outbreak of 2003.			
[21]	Study of environmental evidence of possible airborne transmission of SARS in a hospital ward in Hong Kong in 2003, involving retrospective measurements of ventilation systems, air sampling and computational fluid dynamics simulations to analyses and predict bio-aerosol dispersion in the hospital ward.	High – very confident that the estimated effect is close to the true effect	Evidence that virus-laden aerosol dispersion played a role in the 2003 SARS-CoV outbreak
[22]	Numerical simulation study modelling potential airborne transmission of SARS between apartments - Amoy Gardens outbreak, Hong Kong, 2003	Moderate – the estimated effect is likely to be close to the true effect but there is a possibility that it is substantially different	Evidence that virus-laden aerosol dispersion played a role in the 2003 SARS-CoV outbreak. Evidence that aerosol dispersal is possible between the floors of a building
[23]	Retrospective cohort study of 66 medical students exposed to a SARS inpatient, Hong Kong, 2003. Sample consisted of 16 students with SARS and 50 healthy students. (Study included because it involved inspections and measurements of ventilation system and air flow).	Low – the estimated effect may be substantially different to the true effect	Evidence that virus-laden aerosol dispersion played a role in the 2003 SARS-CoV outbreak
[24]	Epidemiological and fluid mechanics study - the temporal and spatial spread of SARS within a hospital ward, Hong Kong, 2003, was compared with computational fluid mechanics modelling of airborne virus concentrations.	High – very confident that the estimated effect is close to the true effect	Evidence that virus-laden aerosol dispersion played a role in the 2003 SARS-CoV outbreak – the temporal/spatial spread of SARS in this ward was found to be consistent with airborne transmission.
[25]	Epidemiological and fluid dynamics study investigating correlation between the spatial/temporal distrubution of SARS cases in the Amoy Gardens apartment complex, Hong Kong, 2003, with three-dimensional spread of a virus-laden aerosol plume modeled by computational fluid dynamics	Moderate – the estimated effect is likely to be close to the true effect but there is a possibility that it is substantially different	Evidence that virus-laden aerosol dispersion played a role in the 2003 SARS-CoV outbreak. Evidence that aerosol dispersal is possible between buildings
Can live SARS-CoV-2 persist in the air under laboratory conditions? There is high quality evidence that SARS-CoV-2 remains viable in the aerosolised state for several hours, under laboratory conditions.			
[26]	Laboratory-based study investigating the persistence of SARS-CoV-2 under various controlled conditions	High – very confident that the estimated effect is close to the true effect	SARS-CoV-2 remained viable for 3 h in the aerosolised state (median half-life 1.09 h, 95% credible interval 0.64, 2.64), indicating that aerosolised transmission is theoretically possible
Is there evidence for the detection of SARS-CoV-2 RNA in air samples in real-world settings? The quality of the evidence was moderate to very low and the results across studies were inconsistent.			
[27]	Description of infection control measures undertaken during the early stages of the covid-19 pandemic in Hong Kong. Study used real-time PCR methods for detection of SARS-CoV-2 from an air sample. Study also quantified the amount of virus present by reporting viral load/gene copy data.	Low – the estimated effect may be substantially different to the true effect	Air sampler was perpendicularly positioned 10 cm from patient’s chin, and 1000 L air at a rate of 180 L per minute was collected for each culture plate. The patient was instructed to: breathe normally, breathe deeply, say “1, 2, 3” continuously, and cough continuously while putting on and putting off the surgical mask. None of these actions tested positive.
[28]	Hospital-based study in Wuhan, China which tested surface and air samples for presence of SARS-CoV-2 RNA	Moderate – the estimated effect is likely to be close to the true effect but there is a possibility that it is substantially different	This study sampled indoor air and air outlets to detect aerosol exposure. The highest risk area (highest rates of positive tests) were in patient wards and treatment areas, near and downstream from the patients, but positive samples were also found upstream and further away from patients (eg, in doctor’s office), albeit at a lower rate.
[29]	Hospital-based study collecting surface and air samples to test for presence of SARS-CoV-2 RNA, using real-time PCR methods for detection of SARS-CoV-2, Nebraska, USA. Study also quantified the amount of virus present by reporting viral load/gene copy data. Study also attempted to culture live virus from environmental samples.	Low – the estimated effect may be substantially different to the true effect	Study took air samples from isolation rooms (patients, all with mild illness, were present) and hallways. In addition, personal air samplers were worn by study staff during sampling activities. In-room air samples were 63.2% positive. Hallway samples were 66.7% positive. Personal air samplers also tested positive.
[30]	Hospital-based study collecting surface and air samples to test for presence of SARS-CoV-2 RNA, Wuhan, China	Moderate – the estimated effect is likely to be close to the true effect but there is a possibility that it is substantially different	Did not detect SARS-CoV-2 RNA in any of 44 air samples taken
[31]	Cross-sectional study testing surface and air samples for presence of SARS-CoV-2 RNA, in cabins which were occupied by confirmed cases on the Diamond Princess cruise ship, Japan, using real-time PCR methods for detection of SARS-CoV-2. Study also quantified the amount of virus present by reporting viral load/gene copy data. Study also attempted to culture live virus from environmental samples.	Low – the estimated effect may be substantially different to the true effect	Did not detect SARS-CoV-2 RNA in air samples. Most passengers and crew had left the vessel when air sampling was conducted.
[32]	Hospital-based cross-sectional study collecting surface and air samples to test for presence of SARS-CoV-2 RNA, using real-time PCR methods for detection of SARS-CoV-2, London, UK. Study also attempted to culture live virus from environmental samples.	Very low – the estimated effect is very uncertain	Seven clinical areas and a public area of the hospital were sampled. 3-5 air samples were taken from each clinical area. 14/31 (45.2%) of air samples were suspected (12/31, 38.7%) or positive (2/31, 6.4%) for SARS-CoV-2 RNA. Positive or suspected air samples were found in both patient and non-patient areas, however, they were more likely to be found in areas immediately occupied by covid patients. Positive/suspected samples were found in nurses’ station, patient bays, theatres, patient toilets, resus bay (<2 h after last patient), area where CPAP performed. In non-patient areas of the hospital, positive/suspected air samples were found at the main entrance, a public toilet at the main entrance and the staff room.
Is there epidemiological evidence consistent with aerosolised transmission? Evidence from two low quality studies is inconsistent: no strong observational epidemiological evidence for aerosolised transmission.			
[33]	Epidemiological analysis of a disease cluster linked to a choir practice in Washington State, USA	Low – the estimated effect may be substantially different to the true effect	Evidence is potentially consistent with aerosol transmission.
[34]	Epidemiological cross-sectional analysis of data on cases from the outbreak on the Diamond Princess cruise ship, to identify transmission risk factors	Low – the estimated effect may be substantially different to the true effect	After 6 February, when passengers were confined to their cabins, passenger transmission was limited to close contacts (sharing a cabin). The absence of any cross-room transmission among passengers after the quarantine period began supports the hypothesis that transmission was via droplets/fomites and not airborne via the air conditioning system.
Is there evidence from fluid mechanics simulation studies consistent with aerosolised transmission? One high quality numerical simulation study demonstrates that the pattern of secondary infections in a restaurant outbreak is compatible with aerosol transmission.			
[35]	Numerical simulation study (real-scale experiment and computational fluid dynamics simulation), demonstrating probable aerosol transmission of SARS-CoV-2, at an outbreak in a restaurant in Guangzhou, China	High – very confident that the estimated effect is close to the true effect	Study demonstrates that a COVID-19 outbreak in a restaurant in Guangzhou, China, is compatible with aerosol transmission.

To investigate whether aerosolisation of viral particles might actually be occurring, we found six studies which collected and analysed air samples [28-32,39]. Four of the six studies detected SARS-CoV-2 RNA [28,29,32,39]. Whilst the presence of viral RNA can indicate the presence of live virus, it can equally, however, simply indicate the presence of fragmented dead virus, which does not pose an infectivity risk: laboratory culturing methods are required to establish the presence of live virus [40].

We found one study which used tracer gas measurements and computational fluid dynamics simulations to predict the spread of droplets exhaled by the index case in an outbreak linked to a restaurant in Guangzhou, China [35]. The researchers found evidence consistent with aerosolised transmission over short distances within a crowded and poorly ventilated space. We found two observational epidemiological studies reporting evidence relevant to the question of aerosolised transmission. One was an epidemiological investigation report describing a large outbreak in Washington State, USA, linked to a choir practice, which was consistent with aerosolised transmission [33]. However, descriptive epidemiological studies of other outbreaks have failed to find evidence consistent with aerosolised transmission. For example, in an analysis of the outbreak on the Diamond Princess cruise ship researchers argued that the absence of any cross-room transmission once passengers had been quarantined in their cabins supports the hypothesis that transmission was via droplets/fomites and not airborne via the air conditioning system [34].

Taken together, we found evidence that although aerosolised transmission is theoretically possible, we found no conclusive epidemiological evidence of this actually occurring. **Table 3**

**Table 3:** Evidence relating to faecal-oral transmission

Reference	Study type	Overall study quality from critical appraisal	Relevant results
Can SARS-CoV-2 viral RNA be detected in faeces? SARS-CoV-2 RNA was detected in faecal samples in all studies reporting on this. Individual studies were low or very low quality (small case series and case reports, often lacking detail) the consistency of evidence reported across studies suggests that there is evidence for the presence of viral RNA in faeces, although the limitations of the study designs mean that it is not possible to quantify the proportion of cases shedding virus in stool samples.			
[41]	Case report – first case in USA	Low – the estimated effect may be substantially different to the true effect	SARS-CoV-2 viral RNA detected in faecal samples using RT-PCR
[42]	Case series – ten children in Wuhan, China	Low – the estimated effect may be substantially different to the true effect	SARS-CoV-2 viral RNA detected in faecal samples using RT-PCR.
[43]	Case series – 66 convalescent adult patients, Shanghai, China	Low – the estimated effect may be substantially different to the true effect	SARS-CoV-2 viral RNA detected in faecal samples using RT-PCR.
[44]	Case report – ten year old, asymptomatic boy, Zhoushan, China	Low – the estimated effect may be substantially different to the true effect	SARS-CoV-2 viral RNA detected in faecal samples in a ten year old asymptomatic boy using RT-PCR.
[45]	Case series – virological assessment of nine hospitalised cases who acquired infection from the same index case, Germany	Very low – the estimated effect is very uncertain	SARS-CoV-2 viral RNA detected in faecal samples using RT-PCR. 9 adult cases, none severe.
[46]	Case series – real-time RT-PCR results of respiratory and faecal samples from hospitalised patients with COVID-19, Zhuhai, China, throughout the course of their illness and quarantine period.	Very low – the estimated effect is very uncertain	SARS-CoV-2 viral RNA detected in faecal samples using RT-PCR.
[47]	Case series – 14 patients, Jinhua, China	Very low – the estimated effect is very uncertain	SARS-CoV-2 viral RNA detected in faecal samples using RT-PCR.
[48]	Non-systematic review - relationship between COVID-19 and the digestive system	Low – the estimated effect may be substantially different to the true effect	GI symptoms are less common in SARS-CoV-2 than in SARS-CoV or MERS: compared to 30% patients with gastro-intestinal symptoms in SARS and MERS, diarrhoea and vomiting occurred in 5.6% (range of estimates 2-34), and 4.5% (range 1-10) patients of COVID -19, respectively
For how long can viral RNA be detected in faeces? There is evidence that viral RNA may be detectable in stool samples for several weeks after symptom onset and after throat swabs have turned negative. However the quality of the evidence is poor (small case series and case reports, often lacking detail).			
[42]	Case series – ten children in Wuhan, China	Low – the estimated effect may be substantially different to the true effect	5 patients (children) still had SARS-CoV-2 RNA detected in faeces within 1-30 d after illness onset. SARS-CoV-2 faecal samples still tested positive after throat swabs had turned negative.
[43]	Case series – 66 convalescent adult patients, Shanghai, China	Low – the estimated effect may be substantially different to the true effect	At the end of the study, 11 convalescent patients (16.7%) still tested positive for viral RNA from stool specimens. The remaining 55 patients’ stool specimens were negative after a median duration of 11.0 (9.0-16.0) days after symptom onset. SARS-CoV-2 faecal samples still tested positive after throat swabs had turned negative.
[44]	Case report – ten year old, asymptomatic boy, Zhoushan, China	Low – the estimated effect may be substantially different to the true effect	Faecal samples were positive for SARS-CoV-2 RNA at least 26 d after last exposure in a ten year-old asymptomatic boy. SARS-CoV-2 faecal samples still tested positive after throat swabs had turned negative.
[46]	Case series – real-time RT-PCR results of respiratory and faecal samples from hospitalised patients with COVID-19, Zhuhai, China, throughout the course of their illness and quarantine period.	Very low – the estimated effect is very uncertain	Of the 41 (55%) of 74 patients with faecal samples that were positive for SARS-CoV-2 RNA, faecal samples remained positive for a mean of 27 · 9 d (10 · 7) after first symptom onset. SARS-CoV-2 faecal samples still tested positive after throat swabs had turned negative.
Is the presence of viral RNA/live virus in faeces related to the presence of GI symptoms? Evidence from 3 low/very low quality studies consistently showed no relationship between the presence of GI symptoms and detection of viral RNA in stool samples.			
[42]	Case series – ten children in Wuhan, China	Low – the estimated effect may be substantially different to the true effect	None of the ten children in the case series had diarrhoea, but 5 of the 6 who were tested had viral RNA detected in stool samples.
[44]	Case report – ten year old, asymptomatic boy, Zhoushan, China	Low – the estimated effect may be substantially different to the true effect	Faecal samples were positive for SARS-CoV-2 RNA at least 26 d after last exposure in a ten year old asymptomatic boy.
[46]	Case series - Real-time RT-PCR results of respiratory and faecal samples from hospitalised patients with COVID-19, Zhuhai, China, throughout the course of their illness and quarantine period.	Very low – the estimated effect is very uncertain	The presence of gastrointestinal symptoms was not associated with faecal sample viral RNA positivity (*P* = 0.45)
Is there evidence for the aerosolisation of viral particles through toilet flushing? There is low quality evidence for the aerosolisation of viral particles through toilet flushing.			
[29]	Hospital-based study collecting surface and air samples to test for presence of SARS-CoV-2 RNA, using real-time PCR methods for detection of SARS-CoV-2, Nebraska, USA. Study also quantified the amount of virus present by reporting viral load/gene copy data. Study also attempted to culture live virus from environmental samples.	Low – the estimated effect may be substantially different to the true effect	Aerosolisation of viral particles may occur through toilet flushing – detection of SARS-CoV-2 RNA on the floor surrounding toilets used by confirmed cases, which is consistent with aerosolisation of virus particles through toilet flushing
[31]	Cross-sectional study testing surface and air samples for presence of SARS-CoV-2 RNA, in cabins which were occupied by confirmed cases on the Diamond Princess cruise ship, Japan, using real-time PCR methods for detection of SARS-CoV-2. Study also quantified the amount of virus present by reporting viral load/gene copy data. Study also attempted to culture live virus from environmental samples.	Low – the estimated effect may be substantially different to the true effect	Aerosolisation of viral particles may occur through toilet flushing - detection of SARS-CoV-2 RNA on the floor surrounding toilets used by confirmed cases, which is consistent with aerosolisation of virus particles through toilet flushing
[39]	Hospital-based study to measure the concentration of SARS-CoV-2 RNA in aerosols in 2 hospitals in Wuhan, China	Moderate – the estimated effect is likely to be close to the true effect but there is a possibility that it is substantially different	Aerosolisation of viral particles may occur through toilet flushing - the highest concentration of SARS-CoV-2 RNA detected in air samples was in a patient toilet cubicle.
Can live SARS-CoV-2 virus be isolated from faecal samples? We found no evidence for the presence of live virus in stool samples, however this was based on only one, very small study, which attempted unsuccessfully to culture live virus from stool samples, but was successful with lung and throat specimens. This study requires replication with a larger data set.			
[45]	Case series – virological assessment of nine hospitalised cases who acquired infection from the same index case, Germany	Very low – the estimated effect is very uncertain	Live (potentially infectious) virus was isolated from lung and throat specimens but not from stool samples. However, sample size was small (9 adults, none with severe disease). Stool samples were not taken before seroconversion, so this result does not rule out the possibility of faecal-oral transmission.

### What evidence is there for faecal-oral transmission?

Other human coronaviruses can be transmitted via the faeces of infected individuals [25,49,50], so it is important to establish whether SARS-CoV-2 can be transmitted in this way. We reviewed five case series [42,43,45-47], two case reports [41,44], one non-systematic review article [48] and three surface and air sampling studies [29,31,39] (**Table 3**). Emerging evidence suggests that gastro-intestinal (GI) symptoms in SARS-CoV-2 may be the result of viral invasion of ACE2 expressing enterocytes of ileum and colon, as seen with SARS-CoV [51]. However, GI symptoms are less common in SARS-CoV-2 than in SARS-CoV or MERS [48]. All eight articles we reviewed reported detection of SARS-CoV-2 viral RNA in faecal samples using RT-PCR. However, study quality was poor: studies were small and lacked detail and results are difficult to compare because of the different parameters and time frames used, such that estimates of the proportion of adult cases with viral RNA detectable in faeces varied widely. Several studies reported evidence that SARS-CoV-2 faecal samples still tested positive after throat swabs had turned negative [42-44,47]. Several studies reported that the presence of SARS-CoV-2 viral RNA or live virus in faecal samples was unrelated to the presence of gastro-intestinal symptoms [42,44,47]. We also reviewed three studies which collected environmental samples, two in clinical settings [29,39] and one in a cruise ship [31], which suggest that aerosolisation of viral particles may occur through toilet flushing. Two studies highlighted the detection of SARS-CoV-2 RNA on the floor surrounding toilets used by confirmed cases, which is consistent with aerosolisation of virus particles through toilet flushing [29,31]. The highest concentration of SARS-CoV-2 RNA detected in air samples by [39] was in a patient toilet cubicle. However, despite widespread confirmation that viral RNA can be detected in faecal samples, we found no evidence for transmission of the virus by this route. The detection of viral RNA does not mean that live virus is present or that patients are infectious. The only study we found which attempted to isolate live virus was able to isolate infectious virus from samples taken from patients’ throats and lungs, but not from faecal samples, even though these samples had high concentrations of viral RNA [45]. This was a very small study and results require replication. In summary, although viral RNA can be detected in the faeces of cases, we found no evidence of transmission via this route, either through the contamination of surfaces or through aerosolisation.

### What evidence is there regarding the role of ventilation systems in indoor transmission?

We found six experimental and numerical simulation fluid mechanics studies addressing the role of ventilation systems in indoor transmission (**Table 4**). These demonstrate that air currents are responsible for the dispersal of both aerosols and large droplets within buildings, between different rooms and even between different floors [22,52]. Studies show that this dispersal can be amplified by a variety of factors, including ventilation and air conditioning systems [35], differences of temperature between rooms [53] and air currents entering through open windows [54]. However, ventilation systems are also likely to dilute the concentration of viral particles in the air and thereby to play a potential role in decreasing transmission [22,55]. Ventilation systems thus have the potential to decrease virus transmission risk near the source but to increase virus transmission risk further away from the source. However, we found only one study which investigated this question specifically in relation to SARS-CoV-2 [35]. This study used tracer gas experiments and fluid dynamics numerical modelling to predict the location of cases within a poorly ventilated restaurant. Based on this one study alone, which was subject to modelling assumptions and results which were case specific and not clearly generalizable to other indoor environments, the overall quality of the evidence on the role of ventilation systems in indoor transmission of SARS-CoV-2 was judged to be low.

**Table 4:** Evidence relating to the role of ventilation systems in transmission

Reference	Study type	Overall study quality from critical appraisal	Relevant results
Can air currents disperse aerosols and large droplets within buildings? There is moderately strong evidence from two experimental and numerical simulation studies that air currents can readily disperse aerosols and large droplets within buildings. This evidence is not specific to SARS-CoV-2.			
[22]	Numerical simulation study modelling potential airborne transmission of SARS between apartments – Amoy Gardens outbreak, Hong Kong, 2003	Moderate – the estimated effect is likely to be close to the true effect but there is a possibility that it is substantially different	Study showed that during the 2003 SARS outbreak in Hong Kong the ventilation system in the densely populated Amoy Gardens apartment complex contributed to the dispersal of the virus among flats and across different floors and buildings in the complex.
[52]	Experimental fluid mechanics study using tracer gas to study the transmission of airborne particles around an apartment building	Moderate – the estimated effect is likely to be close to the true effect but there is a possibility that it is substantially different	Study showed that an upper apartment can contain up to 7% of the air from the one beneath it, and thus that airborne transmission through ventilation is possible
[25]	Epidemiological and fluid dynamics study investigating correlation between the spatial/temporal distrubution of SARS cases in the Amoy Gardens apartment complex, Hong Kong, 2003, with three-dimensional spread of a virus-laden aerosol plume modeled by computational fluid dynamics	Moderate – the estimated effect is likely to be close to the true effect but there is a possibility that it is substantially different	Study supports the probability of aerosolised transmission of the SARS virus in the outbreak in Amoy Gardens. Virus-laden aerosols were generated in the vertical soil stack of one of the buildings, entering bathrooms via defective floor drain traps. Transportation through and between buildings was then amplified by changes in air temperature/humidity, the suction created by an exhaust fan and the action of wind flows around the building and air flows between apartments.
Can indoor dispersal be amplified by air-conditioning systems? One high quality numerical simulation study found that air conditioners can amplify the dispersal of particles within buildings.			
[35]	Numerical simulation study (real-scale experiment and computational fluid dynamics simulation), demonstrating probable aerosol transmission of SARS-CoV-2, at an outbreak in a restaurant in Guangzhou, China	High – very confident that the estimated effect is close to the true effect	Study showed that there was higher particle concentration in the presence of air recirculation, generated by cold air injected into the room by the air conditioning unit and warm air generated by the people eating in the restaurant
Can indoor dispersal of particles be amplified by differences of temperature between rooms? One high quality experimental study showed that differences in air temperature can cause airflow between rooms. This evidence is not specific to SARS-CoV-2 or even to virus particles – it is based simply on the physical behaviour and properties of particles.			
[53]	Experimental case studies modelling the two-way airflow effect due to temperature difference in indoor air quality	High – very confident that the estimated impact is close to the true impact	Study demonstrates that even small differences of temperature between two rooms can cause a two-way flow between the rooms
Can indoor transmission be amplified by currents entering through open windows? Two moderate quality experimental studies found evidence for the dispersal of particles around buildings amplified by air currents entering through open windows. This evidence is not specific to SARS-CoV-2.			
[54]	Tracer gas experiments to investigate airflow patterns	Moderate – the estimated effect is likely to be close to the true effect but there is a possibility that it is substantially different	Study showed that tracer gas was efficiently distributed from room to room along a building corridor, aided by strong air currents entering through open windows
[52]	Experimental fluid mechanics study using tracer gas to study the transmission of airborne particles around an apartment building	Moderate – the estimated effect is likely to be close to the true effect but there is a possibility that it is substantially different	Study showed that an upper apartment can contain up to 7% of the air from the one beneath it, and thus that airborne transmission through ventilation is possible (ventilation through open windows).
Can ventilation dilute the concentration of viral particles in the air? Two moderate/low quality numerical simulation studies showed that ventilation dilutes the concentration of viral particles close to the source. This evidence is not specific to SARS-CoV-2.			
[22]	Numerical simulation study modelling potential airborne transmission of SARS between apartments – Amoy Gardens outbreak, Hong Kong, 2003	Moderate – the estimated effect is likely to be close to the true effect but there is a possibility that it is substantially different	Study showed that during the 2003 SARS outbreak in Hong Kong the ventilation system in the densely populated Amoy Gardens apartment complex contributed to the dispersal of the virus among flats and across different floors and buildings in the complex. However, study demonstrates that ventilation systems are also likely to decrease the concentration of viral particles in the air: the ventilation system played a fundamental role in mitigating the outbreak by diluting the concentration of virus particles.
[55]	Numerical simulation study investigating the effectiveness of ventilation design for hospital wards in terms of virus removal capacity	Low – the estimated effect may be substantially different to the true effect	Study showed that increasing air exchange rates decreases the risk of contamination in a semi-open hospital ward
What evidence is there for the role of ventilation systems in indoor transmission, specifically in relation to SARS-CoV-2? One high quality numerical simulation study found that the air currents created by an air conditioning unit transported virus particles around a poorly ventilated restaurant, explaining the distribution of subsequent cases.			
[35]	Numerical simulation study (real-scale experiment and computational fluid dynamics simulation), demonstrating probable aerosol transmission of SARS-CoV-2, at an outbreak in a restaurant in Guangzhou, China	High – very confident that the estimated effect is close to the true effect	Study showed that there was higher particle concentration in the presence of air recirculation, generated by cold air injected into the room by the air conditioning unit and warm air generated by the people eating in the restaurant

### What evidence is there regarding the role of plumbing systems in indoor transmission?

There is no direct evidence that SARS-CoV-2 is transmissible via infected faeces; however until this is demonstrated definitively, it is important to understand the potential role of defective plumbing systems. Investigations following the SARS-CoV pandemic provided evidence that defective U-traps played a role in the transmission of SARS-CoV in a large outbreak in the Amoy Gardens residential complex in Hong Kong in 2003. During this outbreak, 321 cases in the apartment complex were linked to faecal-oral transmission [50]. Subsequent simulations have demonstrated that aerosols can be generated in vertical soil stack pipes when toilets are flushed and, if U-traps are defective, can enter a room due to the suction generated by the ventilation system [25,49,56,57]. In this context, contaminated aerosols originating from breath or sewage are more likely to be warmer than the surrounding air, and so are more likely to travel from the lowest to the highest floors of a building than vice versa. The lower the environmental air temperature, the more significant the aerosol transmission from the lowest floors to the highest floors [58]. Evidence is summarised in **Table 5**. In summary, for infectious viruses present in faeces, there is strong real-scale experimental evidence demonstrating the potential for defective plumbing systems to amplify transmission within high-rise buildings, and this is consistent with observational epidemiological evidence. However, as outlined above, we found no evidence for the presence of infectious SARS-CoV-2 in faeces, nor for covid-19 outbreaks amplified through plumbing systems.

**Table 5:** Evidence relating to the role of plumbing systems in transmission

Reference	Study type	Overall study quality from critical appraisal	Relevant results
What is the evidence on the potential of plumbing systems to amplify virus transmission? There is strong evidence from real-scale fluid mechanics field studies and from studies linking epidemiological and fluid mechanics data that defective plumbing systems have the potential to amplify virus transmission, for viruses that can be transmitted through infectious faeces. However, as highlighted above, we found no evidence for faecal-oral transmission of SARS-CoV-2.			
[57]	Real-scale experiment investigating the role of sanitary plumbing systems in the transmission of aerosolised viruses	High – very confident that the estimated effect is close to the true effect	Simulations demonstrate that aerosols can be generated in vertical soil stack pipes when toilets are flushed and can enter a room due to the suction generated by the ventilation system. A functioning U-trap is the only mechanism preventing transportation of aerosolised particles. Yet U-trap failure/depletion can result from a variety of mechanisms and is not unusual. Most of the buildings where defective U-traps have been found are high occupancy and include hospitals.
[49]	Field study investigating foul air and water backflow in a real-scale drainage system	High – very confident that the estimated effect is close to the true effect	Study results confirmed the hypothesis that SARS virus transmission could have occurred through the vertical drainage stack in Amoy Gardens high-rise residential housing complex in Hong Kong, 2003
[56]	Methodological paper on empirical and simulation techniques for the forensic analysis of virus spread via building drainage systems	Moderate – the estimated effect is likely to be close to the true effect but there is a possibility that it is substantially different	Simulations of SARS 2003 Amoy Gardens outbreak demonstrate significant contribution of defective building drainage and ventilation systems – specifically failure of appliance trap seal.
Does environmental temperature affect the transport of viral particles within buildings? There is evidence that aerosol transmission from low to high floors is greater the lower the environmental temperature; however this was based on one very low quality study.			
[58]	Numerical simulation and field experiment investigating airborne transmission within a high rise building	Very low – the estimated effect is very uncertain	Contaminated aerosols originating from breath or sewage are more likely to be warmer than the surrounding air, and so are more likely to travel from the lowest to the highest floors of a building than vice versa. The lower the environmental air temperature, the more significant the aerosol transmission from the lowest floors to the highest floors

### What evidence is there regarding transmission via different indoor surfaces (materials and specific objects)?

We identified 13 studies investigating the transmission potential of different materials, surfaces and objects in indoor environments, summarised in **Table 6** [26,27,29-32,59-65]. The length of time SARS-CoV-2 remains viable on surfaces depends on the type of surface and the environmental conditions. Experimental evidence from tightly controlled laboratory studies indicates that the virus survives better on smooth, non-porous surfaces, at low temperatures and in damp conditions [26,59-61]. It can also survive under acidic conditions, such as the stomach [61]. Although there is general agreement among studies that the virus survives for longer on smooth surfaces and at lower temperatures, estimates of precisely how long it can survive on different surfaces vary considerably among studies, likely because of differences in experimental conditions. Furthermore, these studies are silent on the infectious dose and do not quantify the risk of transmission associated with touching different objects and surfaces. It is also important to note that studies conducted under strict laboratory conditions are not directly applicable to real-world contexts, so these findings must be triangulated with studies collecting and analysing environmental samples. Several studies reported detecting viral RNA on a wide range of high-touch objects; however in low quantities [27,29-32,62,63]. As highlighted above, viral RNA can be either live virus, which poses an infectivity risk, but equally it can be fragmented dead virus which does not have the ability to cause infection. We found three studies which attempted to culture live virus from environmental samples, all with inconclusive or negative results [29,31,32]. Epidemiological evidence on this question is inconclusive because it is difficult to distinguish from descriptive epidemiological data alone between fomite and droplet transmission. A contact tracing report on a church outbreak in Singapore found that one of the three secondary cases had no direct contact with the presumed index cases (a couple visiting from China), but occupied the seat that one of them had vacated [65]. However, this was a small, very low quality study and whilst this evidence is consistent with transmission via touching a contaminated object, it is also consistent with airborne transmission.

**Table 6:** Evidence relating to fomite transmission

Reference	Study type	Overall study quality from critical appraisal	Relevant results
What is the evidence from laboratory studies on the length of time SARS-CoV-2 survives on different surfaces? High quality evidence from laboratory studies suggests that live virus persists for days to smooth, non-porous surfaces, compared to hours on rough/porous surfaces, although not necessarily at infectious dose (NB, study conducted under strictly controlled laboratory conditions, not directly applicable to real-life conditions).			
[59]	Laboratory based study investigating the stability of SARS-CoV-2 in different environmental conditions.	Low – the estimated effect may be substantially different to the true effect	SARS-CoV-2 was more stable on smooth than rough surfaces. No infectious virus could be detected on day 4 (glass and banknote) or day 7 (stainless steel and plastic).
[60]	Laboratory based study investigating the survival rates of infectious SARS-CoV-2 on common surfaces (cotton, glass, steel, vinyl, paper and polymer banknotes) at three different temperatures (20°C, 30°C, and 40°C) with no exposure to UV light (known to rapidly deactivate the virus) and humidity controlled at 50%.	High – very confident that the estimated effect is close to the true effect	The virus survived for considerably longer on smooth (non-porous) surfaces. Survival times in this study were considerably longer than in the studies by van Doremalen and Chin, likely because of differences in experimental conditions: the researchers were able to recover live virus after 28 d from the smooth surfaces, although not necessarily at infectious dosages.
[26]	Laboratory-based study investigating the persistence of SARS-CoV-2 under various controlled conditions	High – very confident that the estimated effect is close to the true effect	The researchers found that the virus persisted for up to 72 h after application to plastic (median half-life 6.81 h, 95% credible interval 5.62, 8.17) and up to 48 h after application to stainless steel (median half-life 5.63 h, 95% credible interval 4.59, 6.86). The virus was found to be more stable on these surfaces than on copper (median half-life 0.774 h, 95% credible interval 0.427, 1.19) and cardboard (median half-life 3.46 h, 95% credible interval 2.34, 5). After 4 h, no viable SARS-CoV-2 was detectable on copper and after 24 h no viable SARS-CoV-2 was detectable on cardboard.
What is the evidence from laboratory studies on how long SARS-CoV-2 can survive at different temperatures? A high quality laboratory study found that the virus persists longer at lower temperatures, surviving on common surfaces for days at 20°C, compared less than 24 h at 40°C (n.b. study conducted under strictly controlled laboratory conditions, not directly applicable to real-life conditions).			
[60]	Laboratory based study investigating the survival rates of infectious SARS-CoV-2 on common surfaces (cotton, glass, steel, vinyl, paper and polymer banknotes) at three different temperatures (20°C, 30°C, and 40°C) with no exposure to UV light (known to rapidly deactivate the virus) and humidity controlled at 50%.	High – very confident that the estimated effect is close to the true effect	Study found that the virus survived for longer at lower temperatures. The researchers estimated half lives of between 1.7 and 2.7 d at 20°C, reducing to a few hours at 40°C. They estimated that viable virus could be detected up to 28 d at 20°C from common surfaces such as glass, stainless steel and banknotes (both paper and polymer). Infectious virus survived less than 24 h at 40°C on some surfaces.
What is the evidence from laboratory studies on how long SARS-CoV-2 can survive in wet vs dry conditions? One low quality study found that the virus survived for longer in wet compared to dry conditions (n.b. study conducted under strictly controlled laboratory conditions, not directly applicable to real-life conditions).			
[61]	Laboratory based study using a strain from the nasal-pharyngeal swab of a clinically confirmed COVID-19 patient in Shanghai, investigating the stability of SARS-CoV-2 in wet, dry and acidic conditions at room temperature. The researchers measured the stability of SARS-CoV-2 in wet (in 100 μL culture medium) and dry (10 μL supernatant on filter paper) environments at room temperature (22°C) each day for 7 d, as well as its stability under acidic conditions to mimic the gastric environment (pH2.2)	Low – the estimated effect may be substantially different to the true effect	Although the virus survived for 3 d in both the wet and dry environments, the dry environment was less favourable for virus survival. Viable virus was not observed after 4 d in either the wet or dry condition.
What is the evidence from laboratory studies on how long SARS-CoV-2 can survive in acidic conditions? One low quality study found that the virus survived for at least an hour under acidic conditions mimicking the gastric environment (n.b. study conducted under strictly controlled laboratory conditions, not directly applicable to real-life conditions).			
[61]	Laboratory based study using a strain from the nasal-pharyngeal swab of a clinically confirmed COVID-19 patient in Shanghai, investigating the stability of SARS-CoV-2 in wet, dry and acidic conditions at room temperature. The researchers measured the stability of SARS-CoV-2 in wet (in 100 μL culture medium) and dry (10 μL supernatant on filter paper) environments at room temperature (22°C) each day for 7 d, as well as its stability under acidic conditions to mimic the gastric environment (pH2.2)	Low – the estimated effect may be substantially different to the true effect	The researchers found that the virus tolerated an acidic environment, surviving for at least one hour under acidic conditions mimicking the gastric environment.
What is the evidence from environmental swab studies on the detection of SARS-CoV-2 viral RNA on surfaces/objects? Five low, one very low and two moderate quality studies reported on the detection of viral RNA on a range of different high touch objects and surfaces (eg, bed sheets/pillows, doorknobs, phones, computers). RNA was also detected around toilet areas. One study found no RNA but swabs were taken after cleaning.			
[27]	Description of infection control measures undertaken during the early stages of the covid-19 pandemic in Hong Kong. Study used real-time PCR methods for detection of SARS-CoV-2 from surface samples. Study also quantified the amount of virus present by reporting viral load/gene copy data.	Low – the estimated effect may be substantially different to the true effect	Study found low levels of viral material. Thirteen environmental samples were taken from surfaces/objects in the room of an infected patient (bench, bedside rail, locker, bed table, alcohol dispenser, and window bench), of which one tested positive (7.7%). The surface that tested positive was the window bench.
[28]	Hospital-based study in Wuhan, China which tested surface and air samples for presence of SARS-CoV-2 RNA	Moderate – the estimated effect is likely to be close to the true effect but there is a possibility that it is substantially different	Positive swabs were most concentrated in contaminated areas (ICU and ward housing covid patients). Highest rates of positive swabs in contaminated areas were on frequently touched objects – specifically computer mice (75%), refuse bins (60%), bedrails (43%). Positive rates were also found for floor swabs (70%) – droplets and aerosols eventually land on the floor – and on the soles of staff shoes. Half of the shoe soles of ICU staff tested positive and 100% of swabs taken from the floor of the pharmacy tested positive, despite no patients visiting the pharmacy. Levels of contamination in other parts of the hospital were much lower (8% in semi-contaminated area and zero in clean area)
[62]	Letter to the editor describing the effectiveness of hospital environmental cleaning procedures in preventing transmission from an infected case, Japan. Study used real-time PCR methods for detection of SARS-CoV-2 on 15 environmental samples from rooms occupied by an infected patient, with samples collected after thorough cleaning of the area.	Low – the estimated effect may be substantially different to the true effect	This study took swabs from 15 surfaces in an area occupied by an infected case, after thorough environmental cleaning. Researchers did not detect any viral RNA, which provides evidence on the effectiveness of cleaning to reduce transmission.
[63]	Study collecting surface samples to test for presence of SARS-CoV-2 RNA, using real-time PCR methods for detection of SARS-CoV-2, in 2 rooms occupied by 2 pre-symptomatic confirmed cases in a quarantine hotel, China	Low – the estimated effect may be substantially different to the true effect	Study tested 22 samples from a range of objects/surfaces (door handle, light switch, tap, thermometer, television remote, pillow cover, duvet cover, sheet, towel, bathroom door handle, and toilet seat and flushing button). Eight (36%) tested positive. Samples collected from a tap, sheet, duvet cover, pillow cases from both rooms, and towel tested positive. The samples from the pillow case and sheet belonging to one of the cases had a high viral load.
[29]	Hospital-based study collecting surface and air samples to test for presence of SARS-CoV-2 RNA, using real-time PCR methods for detection of SARS-CoV-2, Nebraska, USA. Study also quantified the amount of virus present by reporting viral load/gene copy data. Study also attempted to culture live virus from environmental samples.	Low – the estimated effect may be substantially different to the true effect	Study sampled three categories of surfaces: common room surfaces (ventilation grates, tabletops, and window ledges), personal items (mobile phones, exercise equipment, television remotes, computers, iPads, reading glasses, medical equipment), and toilets. 76.5% of all personal items sampled tested positive. Mobile phones were 83.3% positive, TV remote controls were 64.7% percent positive. Samples of the toilets in the room were 81.0% positive. 80.4% of all room surfaces were positive, including 75.0% of bedside tables and bed rails and 81.8% of window ledges. All five floor samples and 4/5 ventilation grate samples tested positive. The presence of viral RNA on the floor under patient beds and on window ledges is suggestive of turbulent air currents transporting viral material.
[30]	Hospital-based study collecting surface and air samples to test for presence of SARS-CoV-2 RNA, using real-time PCR methods for detection of SARS-CoV-2 from surface samples, Wuhan, China	Moderate – the estimated effect is likely to be close to the true effect but there is a possibility that it is substantially different	Almost 25% of samples taken in medical areas were positive, compared to <4% of samples from living areas, a difference which was statistically significant (*P* < 0.05). Positive rates were 25.00% and 37.50% for the general isolation ward and intensive care unit, respectively (not significantly different, *P* = 0.238). The top 5 sampling sites with a positive rate in medical areas were beepers (50.00%), water machine buttons (50.00%), elevator buttons (42.86%), computer mice (40.00%), and telephones (40.00%).
[31]	Cross-sectional study testing surface and air samples for presence of SARS-CoV-2 RNA, in cabins which were occupied by confirmed cases on the Diamond Princess cruise ship, Japan, using real-time PCR methods for detection of SARS-CoV-2. Study also quantified the amount of virus present by reporting viral load/gene copy data. Study also attempted to culture live virus from environmental samples.	Low – the estimated effect may be substantially different to the true effect	Of 601 samples tested, SARS-CoV-2 RNA was detected from 58 samples (10%). SARS-CoV-2 RNA was detected from case-cabins but not from non-case-cabins. It was detected in only one sample from common areas of the ship. It was most often detected on the floor around toilet in the bathroom (39%, 13/33) and bed pillow (34%, 11/32). There was no difference in the detection proportion between cabins for symptomatic (15%, 28/189) and asymptomatic cases (21%, 28/131) (*P* > 0.05). Viral RNA was present on highly touched surfaces in cabins such as the room phone, TV remote and the doorknob before and after spraying with 5% hydrogen peroxide solution, indicating that wiping surfaces may be more effective at disinfection than only spraying surfaces. High virus detection was also observed on bed pillows.
[64]	Hospital-based study collecting samples from surfaces and objects to test for presence of SARS-CoV-2 RNA, using real-time PCR methods for detection of SARS-CoV-2, Wuhan, China.	Moderate – the estimated effect is likely to be close to the true effect but there is a possibility that it is substantially different	Of the 626 samples, 13.6% were positive for SARS-CoV-2 RNA. Viral RNA was detected on a wide range of objects and surfaces. The most contaminated were self-service printers used by patients to print out their test reports (20%), desktop/keyboard (16.8%), doorknob (16.0%).
[32]	Hospital-based cross-sectional study collecting surface and air samples to test for presence of SARS-CoV-2 RNA, using real-time PCR methods for detection of SARS-CoV-2, London, UK. Study also attempted to culture live virus from environmental samples.	Very low – the estimated effect is very uncertain	Study detected viral RNA, but no live virus, in both clinical and public areas of the hospital, although this was significantly more likely to be found in areas of the hospital occupied by covid-19 patients (OR = 0.5, 95% CI = 0.2-0.9, *P* = 0.025). They detected viral RNA on 114/218 (52.3%) of surfaces. These swabs were taken from several different objects, including chairs, computer keyboards and alcohol hand sanitiser dispensers.
Can live SARS-CoV-2 be cultured from environmental samples? Three low/very low quality studies attempted to culture live virus from RNA samples taken from environmental surfaces. One study was unable to culture live virus and the results of the other two were inconclusive.			
[29]	Hospital-based study collecting surface and air samples to test for presence of SARS-CoV-2 RNA, using real-time PCR methods for detection of SARS-CoV-2, Nebraska, USA. Study also quantified the amount of virus present by reporting viral load/gene copy data. Study also attempted to culture live virus from environmental samples.	Low – the estimated effect may be substantially different to the true effect	Results on the presence/absence of live virus were inconclusive.
[31]	Cross-sectional study testing surface and air samples for presence of SARS-CoV-2 RNA, in cabins which were occupied by confirmed cases on the Diamond Princess cruise ship, Japan, using real-time PCR methods for detection of SARS-CoV-2. Study also quantified the amount of virus present by reporting viral load/gene copy data. Study also attempted to culture live virus from environmental samples.	Low – the estimated effect may be substantially different to the true effect	Results on the presence/absence of live virus were inconclusive – possible reasons suggested for inconclusive results on the culturing of live virus include transport time to the laboratory, methodological errors.
[32]	Hospital-based cross-sectional study collecting surface and air samples to test for presence of SARS-CoV-2 RNA, using real-time PCR methods for detection of SARS-CoV-2, London, UK. Study also attempted to culture live virus from environmental samples.	Very low – the estimated effect is very uncertain	Study was unable to culture live virus from samples. Possible reasons suggested for the inability to culture live virus include low RNA levels in the samples, or virus that is infectious but not culturable in the laboratory.
What is the evidence for fomite transmission from observational epidemiological studies? One very low quality epidemiological study described a scenario consistent with fomite transmission.			
[65]	Epidemiological outbreak study analysing contact tracing data on an outbreak linked to a church service in Singapore.	Very low – the estimated effect is very uncertain	Study found that one of the three secondary cases did not have direct contact with the presumed index cases, but occupied the same seat as one of them at a prayer meeting directly following the service but not attended by the index cases.

### What evidence is there for the transmission of COVID-19 in indoor residential settings?

**Figure 2:**
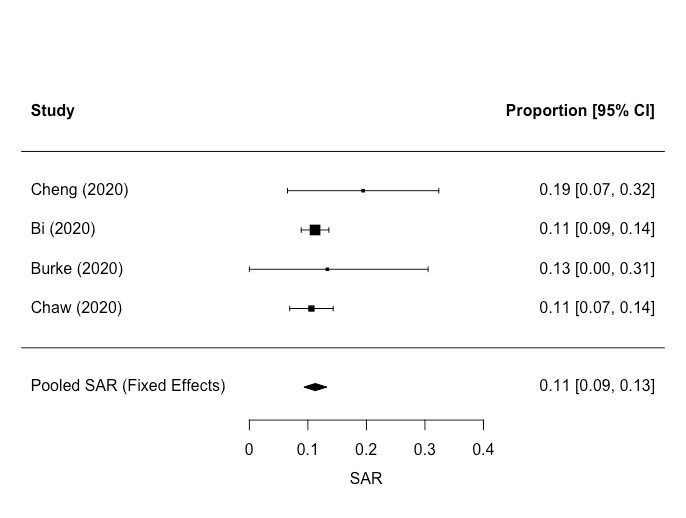
Forest plot – pooled estimate of household secondary attack rate (SAR). I^2^ = 0.00%, Q(df = 3) = 1.72, *P* = 0.63.

Eight studies included data on transmission in residential settings. Four of these reported on household transmission [66-69], providing data on secondary attack rates (SAR, defined as the probability that an infection occurs among susceptible people within a specific group, such as a household or close contacts [70] (SARs)) (**Table 7**). We conducted a meta-analysis of the SARs for these four studies. The pooled SAR for people living in the same household was 11% (95% CI = 9, 13) (**Figure 2**). We found four studies reporting data for estimating SARs amongst residents in communal living environments [34,71-73]. SARs for residents in these settings are shown in **Table 8**. SARs for staff working in these settings are shown separately in Table 9. These studies involved very different types of population (elderly nursing home residents, passengers on a cruise ship and people experiencing homelessness), so it was not appropriate to conduct a meta-analysis. The SARs for people living in communal settings were significantly higher than the SARs for households. The quality of epidemiological evidence for transmission in residential and communal settings was poor.

**Table 7:** Secondary Attack Rates (SARs) within households

Study ID	Date of investigation	Country	Study quality	Context	n contacts	n cases	SAR (%)	95% confidence interval
[69]	Jan-Feb 2020	Taiwan	Very low – the estimated impact is very uncertain	Household – living in the same house with index case	36	7	19	7, 32
[66]	Jan – Feb 2020	China	Low – the estimated effect may be substantially different to the true effect	Household – living in the same house with index case	686	77	11	9, 14
[67]	Jan – Feb 2020	USA	Very low – the estimated impact is very uncertain	Household, defined as family or friends spending at least one night in the same residence as case during presumed infectious period	15	2	13	0, 31
[68]	March – April 2020	Brunei	Low – the estimated effect may be substantially different to the true effect	Household transmission	264	28	11	7, 14
Pooled estimate					1001	114	11	9, 13

**Table 8:** Secondary attack rates among residents in communal or assisted living contexts

Study ID	Date of investigation	Country	Study Quality	Context	n contacts	n cases	SAR (%)	95% confidence interval	Comments
[71]	Feb 2020	USA	Very low – the estimated effect is very uncertain	Care home for long-term residents requiring skilled nursing care	130	81	62.3	54.0, 70.6	Number of residents given is “approximately 130”
[72]	Mar 2020	USA	Low – the estimated effect may be substantially different to the true effect	Sheltered housing for the elderly. Comprises 83 separate apartments and multiple corridors and communal dining, library and activity areas. 45 of the apartments are for independent living, 38 for assisted living (people having a daily home help for assistance with activities of daily living/medication). This is not a nursing home.	80	3	3.8	0.0, 7.9	A high proportion of both residents and staff reported symptoms but tested negative. This may have been due to recall bias, given the high levels of anxiety about COVID-19, but it may also indicate false negative test results
[73]	March – April 2020	USA	Low – the estimated effect may be substantially different to the true effect	3 affiliated overnight and day centres for homeless people (comprising a 24-h shelter serving up to 40 men and 10 women (A); an overnight shelter housing up to 110 men in 2 main rooms (B); an overnight shelter housing up to 100 men in 2 main rooms (C). Shelters have onsite indoor bathrooms with sinks and soap. Residents from shelters B and C used shelter A’s day centre services.	195	35	18.0	12.6, 23.3	
[34]	Feb 2020	Japan	Low – the estimated effect may be substantially different to the true effect	Cruise ship – passengers of Diamond Princess cruise ship – quarantined off Japan due to outbreak	2666	522	19.6	17.6, 20.3	Figures are as of 5 March 2020; however some cases developed later

**Table 9:** Secondary attack rates in workplaces

Study ID	Date of investigation	Country	Context	n contacts	n cases	SAR (%)	95% confidence interval	Comments
[71]	Feb 2020	USA	Care home for long-term residents requiring skilled nursing care – staff	170	34	20.0	14.0, 26.0	
[72]	Mar 2020	USA	Sheltered housing for the elderly – staff. Comprises 83 separate apartments and multiple corridors and communal dining, library and activity areas for assisted and independent living. This is not a nursing home.	62	2	3.2	0.0, 7.6	A high proportion of both residents and staff reported symptoms but tested negative. This may have been due to recall bias, given the high levels of anxiety about COVID-19, but it may also indicate false negative test results.
[73]	March – April 2020	USA	Homeless shelter – staff	38	8	21.0	8.1, 34.0	
[34]	Feb 2020	Japan	Cruise ship – crew of Diamond Princess cruise ship – quarantined off Japan due to outbreak	1045	144	13.8	11.7, 15.9	Figures are as of 5 March 2020; however some cases developed later.

### What evidence is there for the transmission of COVID-19 in indoor workplaces?

Six studies reported on transmission among workers or at workplaces, where details were provided about the nature of the work and workplace. These were: care home workers [71], cruise ship crew [34], staff at a shelter for people experiencing homelessness [73], staff at an assisted and independent living community for the elderly [72], workers at meat/poultry processing plants [74] and shop workers [65]. Four of these studies provided data for the estimation of SARs among staff (**Table 9**). All four of the workplaces shown in **Table 9** are also places of residence (SARs for residents are shown in **Table 8**). SARs for staff and residents were not significantly different in the assisted and independent living community or in the shelter; however, SARs were significantly higher for residents than for staff on the cruise ship (*P* = 0.000017) and in the care home (*P* < 0.00001). We found two workplace studies which did not present sufficient data to estimate SARs but nevertheless provide insight into workplace transmission (**Table 10**). A CDC paper reporting on outbreaks in meat processing plants across the USA [74] identified a range of key drivers. These included difficulty in maintaining the 2 m social distance on the production line at break times and while entering/exiting the facility; difficulty implementing covid-19-specific disinfection guidelines; socioeconomic challenges related to poverty, such as people continuing to work whilst ill, especially where attendance is incentivised and workers living in overcrowded, multigenerational households; communication challenges such as the inaccessibility of health and safety training to non-English speakers and to non-literate workers; sharing of transportation to work; and adherence to correct usage of face coverings. Another driver may be that factories are noisy environments, where people may have to shout, thus transmitting droplets over longer distances. Results of a small contact tracing study of an outbreak in Singapore connected with a shopping trip of a group of tourists from China points to close and prolonged interactions with a case as a driver of transmission [65]. Overall, the quality of the epidemiological evidence on workplace transmission was poor. There is considerable variability in workplace contexts, making it difficult to synthesise conclusions across settings, and detail is often lacking as to potential transmission mechanisms.

**Table 10:** Details of studies providing insights into risk factors for workplace transmission

Reference	Study type	Overall study quality from critical appraisal	Relevant results
[74]	Centers for Disease Control (CDC) report on workplace outbreaks in meat and poultry processing facilities across the USA.	Low – the estimated impact may be substantially different to the true impact	The article presents data from 17 of 23 US states reporting at least one such outbreak, expressing the number of cases in each state as a proportion of all meat and poultry workers employed in the state. In other words, the denominator includes workers in facilities which have not experienced an outbreak, thus under-estimating the impact of such an outbreak on an individual facility. By April 2020 there had been a total of 4913 cases in a total workforce of 130578 in the 17 states who provided full data (3.8%, 95% CI = 3.7, 3.9). The study highlighted socioeconomic factors linked to poverty as key drivers.
[65]	Epidemiological contact tracing study of an outbreak in Singapore connected with the visit of a tour group of around 20 tourists from China to a complementary health products shop and to a jewelry shop.	Very low – the estimated impact is very uncertain	Four assistants in the complementary health products shop and one assistant in the jewellery shop were subsequently confirmed to have COVID-19, after the tourists spent a prolonged period in the shops. In the complementary health products shop, there was close physical contact between some of the tourists and the shop workers.

### What evidence is there for the transmission of COVID-19 in other indoor settings (social, community, leisure, religious, public transport)?

We found three epidemiological studies reporting on transmission related to social, religious, community or leisure settings and providing sufficient data to estimate SARs (**Table 11**). Two studies report on a total of three outbreaks related to religious gatherings or churches [65,68]. One study investigated evidence for transmission in a clinic waiting room [67].

**Table 11:** Details of studies providing insights into risk factors for transmission in other indoor settings

Reference	Study type	Overall study quality from critical appraisal	Relevant results
[68]	Epidemiological analysis of contact tracing data linked to an outbreak centred on an Islamic religious gathering (Tablighi Jama’at) in Kuala Lumpur, Malaysia and attended by 75 citizens of Brunei, of whom 19 became ill. There were a further 52 additional secondary/subsequent cases in Brunei, bringing the cluster size to 71. Study investigates environmental, behavioural and host risk factors for transmission.	Low – the estimated effect may be substantially different to the true effect	Study reports SARs for outbreaks related to the Tablighi Jama'at religious gathering in Malaysia and a subsequent similar gathering in Brunei. Both were extended, communal overnight gatherings. Estimated SARs were 25.3% (95% CI = 15.5, 35.2) and 14.8% (95% CI = 5.3, 24.3) respectively.
[65]	Epidemiological outbreak study analysing contact tracing data on an outbreak linked to a church service in Singapore.	Very low – the estimated effect is very uncertain	The presumed index cases were a couple visiting from China who had attended a service at the church. Three of the 142 contacted attendees at the service subsequently tested positive for SARS-CoV-2 (SAR 2.1; 95% CI = 0, 4.4).
[67]	Epidemiological contact tracing study of the first 9 travel-related cases identified in the USA, and 338 of their close contacts – follow up of close contacts to identify transmission risk factors.	Very low – the estimated effect is very uncertain	Study followed up 95 people who spent time in clinic waiting rooms with affected individuals. No cases were detected.

Estimated SARs ranged from 2.1% at a church service in Singapore [65] to 25.3% at an extended, overnight religious gathering in Malaysia [68]. The study investigating transmission in clinic waiting rooms followed up 95 people who spent time in clinic waiting rooms with affected individuals in USA. No cases were detected [67]. The quality of this observational evidence on transmission in social/community settings was very poor and there was considerable heterogeneity of contexts and variability in the results.

### Do particular activities convey greater risk (eg, shouting, singing, eating together, sharing bedrooms)?

Different activities involve the emission of different numbers of respiratory droplets. Evidence from fluid mechanics experiments shows that the number of droplets ejected increases in the order: breathing, heavy breathing, speaking, singing, coughing, sneezing. There is a very significant (orders-of-magnitude) difference in the numbers of droplets emitted between each of these levels and the next [75-79]. There is also evidence that pronouncing some vowel sounds results in the emission of more droplets than others; however these risk differences are relatively small compared to the risks between, for example, coughing and singing [80]. Although the physical properties and behaviour of droplets emitted via different mechanisms are well characterised, it is not possible directly to compare the risks of transmission associated with heavy breathing with those associated with coughing or sneezing. This is because whilst breathing is a continuous activity, coughing and sneezing are discrete events and are thus not directly comparable in terms of risk level. Different activities result in the emission of droplets of different sizes (for example, small droplets are emitted during breathing, and large droplets when sneezing). Thus droplets emitted by these different activities will be associated with different transmission mechanisms. A final point to consider is that droplets emitted through these different mechanisms are generated in different parts of the respiratory system, and thus, are likely to have different viral loads.

**Table 12** details four descriptive epidemiological studies which describe transmission via daily living activities among people living together in households [66-69]. The results of these studies are consistent with the hypothesis that close and prolonged contact through activities such as sharing beds, bathrooms, eating together, face to face contact and spending time in the car together are likely to increase the risk of transmission. Again, however, the quality of individual studies was poor or very poor and there is insufficient evidence to evaluate the relative risk of specific activities or behaviours from these studies.

**Table 12:** Evidence relating to the risks of transmission associated with specific activities or behaviours

Reference	Study type	Overall study quality from critical appraisal	Relevant results
What are the physical properties and behaviour of droplets and aerosols ejected while breathing, speaking, singing, coughing, sneezing? Three high and one moderate quality experimental studies conducted under carefully controlled conditions found that: loud speech emits a higher rate of particles than quiet speech, coughing emits more, smaller, faster and more concentrated droplets than speaking.			
[79]	Experimental study in which human subjects repeatedly said the vowel sound in the word “saw” but at different amplitudes. The volume of particles emitted was measured.	High – very confident that the estimated effect is close to the true effect	This study found that the rate of particle emission during normal human speech correlated positively with the volume (loudness) of the speech.
[75]	Laboratory experimental study which measured expired droplets from human subjects coughing and speaking (counting from 1 to 100). Expiration velocities and droplet size distributions were measured.	High – very confident that the estimated effect is close to the true effect	The average expiration air velocity was 11.7 m/s for coughing and 3.9 m/s for speaking. The geometric mean diameter of droplets from coughing was 13.5 μm and it was 16.0 μm for speaking . The estimated total number of droplets expelled ranged from 947 to 2085 per cough and 112-6720 for speaking. The estimated droplet concentrations for coughing ranged from 2.4 to 5.2 cm^3^ per cough and 0.004- to 0.223 cm3 for speaking.
[77]	Experiment measuring the number and size of respiratory droplets emitted during speaking and coughing.	Moderate – the estimated effect is likely to be close to the true effect but there is a possibility that it is substantially different	Study did not find a big difference in the size distribution of droplets produced between coughing and talking, although this may be because healthy volunteers were used. More small droplets were produced during coughing than during speech.
[78]	Experimental study which measured the size and number of droplets emitted by human subjects whilst coughing in order to characterize the human cough aerosol pattern	High – very confident that the estimated effect is close to the true effect	The study found that coughs generated droplets ranging from 0.1-900 microns in size. Droplets of less than one-micron size represent 97% of the total
Do some sounds result in the emission of more droplets than others? One high quality experimental study conducted under carefully controlled conditions found differences in the numbers of particles emitted by different vowel sounds.			
[80]	Experimental study measuring the emission rate of respiratory aerosols in human subjects when voicing different sounds, both in normal speech and as isolated sounds.	High – very confident that the estimated effect is close to the true effect	Study found that certain sounds are associated with significantly higher particle production; for example, the vowel sound in the words “need,” and “sea” produces more particles than the vowel sound in the words “saw,” or “hot”) or the vowel sounds in the word “blue,” or “mood”). Consonants such as p, t, k, b, d, g emit more particles than consonants such as f or sounds such as th.
Is there observational epidemiological evidence for transmission via daily living activities within households? The overall quality of the evidence is low to very low. We found two low and two very low quality epidemiological studies. One found evidence that transmission was associated with daily living activities such as travelling or eating meals together. One found evidence of transmission to spouses but not to other household members. One found significantly higher transmission to spouses than to other relatives. One found higher transmission to relatives in the same household compared to relatives living apart, although differences were not significant.			
[66]	Epidemiological analysis of symptomatic surveillance and contact tracing data for 391 SARS-CoV-2 cases and 1286 controls identified from 14 January – 12 February 2020, Shenzhen, China. Purpose of study was to estimate metrics of transmission and investigate transmission risk factors. The researchers followed up cases and close contacts for 14 d and then retested. Close contacts were defined as people living in the same apartment, sharing a meal, travelling together, or interacting socially with the index case from 2 d before the onset of symptoms.	Low – the estimated effect may be substantially different to the true effect	A multivariate regression analysis estimated the OR for household contacts as 6.3 (95% CI = 1.5, 26.3), travelling together 7.1 (95% CI = 1.4, 34.9) and eating meals together 7.13 (95% CI = 0.73, 69.32). The OR for having contact “often” with the index case (compared to having rare or moderate contact) was 8.8 (95% CI = 2.6, 30.1).
[67]	Epidemiological contact tracing study of the first 9 travel-related cases identified in the USA, and 338 of their close contacts – follow up of close contacts to identify transmission risk factors.	Very low – the estimated effect is very uncertain	2 cases resulted from household transmission, both in the spouses of cases. The authors suggest that daily living activities such as sharing beds, bathrooms, eating together, face to face contact and spending time in the car together are likely to increase the risk of transmission. Family members cohabiting during case isolation were advised where possible to use separate bedrooms and bathrooms, limit time in same room and affected family members were advised to wear a mask when in the same room as others. The study reported strong compliance in general with these measures, with some evidence that there was higher compliance with isolation measures and less time spent with affected family members in households where there was no transmission.
[68]	Epidemiological analysis of contact tracing data linked to an outbreak centred on an Islamic religious gathering (Tablighi Jama’at) in Kuala Lumpur, Malaysia and attended by 75 citizens of Brunei, of whom 19 became ill. There were a further 52 additional secondary/subsequent cases in Brunei, bringing the cluster size to 71. Study investigates environmental, behavioural and host risk factors for transmission. The study also investigated attack rates for different relationships living together in households	Low – the estimated effect may be substantially different to the true effect	The study found that the highest secondary attack rate was amongst spouses, at 41.94% (95% CI = 26.42, 59.24). This compares with 14.12% (95% CI = 8.27, 23.08) for children and 2.03% (95% CI = 0.69, 5.79) for other relatives (parents, siblings, grandparents, housekeepers, etc.).
[69]	Epidemiological analysis of contact tracing data to understand transmission dynamics and estimate the infectious period.	Very low – the estimated effect is very uncertain	This study compared secondary attack rates in household members with non-household family members. The secondary attack rate in people living in the same household was 19.44% (95% CI = 9.75, 35.02) compared to 10.64% (95% CI = 4.63, 22.6) in relatives living apart, although the difference is not significant.
Is there observational epidemiological evidence for transmission via daily living activities within communal residential settings? The quality of the evidence is low/very low but suggests higher transmission in more communal compared with more separate residential settings.			
[71]	Epidemiological report on an outbreak in a residential elderly care facility in Washington State, USA (resulting in 81 residents, 34 staff members, and 14 visitors becoming ill)	Very low – the estimated effect is very uncertain	Report recommends restricting resident movement, group activities and visitation and enforcing physical distancing to avoid outbreaks.
[72]	Epidemiological report on an outbreak in an independent living facility for the elderly (sheltered housing) in Seattle, Washington State, USA (resulting in 4 residents testing positive)	Low – the estimated effect may be substantially different to the true effect	Transmission rates were striking low in this independent and assisted living facility, compared to outbreaks in nursing homes, which are more communal living environments.
[73]	Epidemiological report of an outbreak in 3 affiliated overnight and day centres for homeless people (comprising a 24-h shelter serving up to 40 men and 10 women (A); an overnight shelter housing up to 110 men in 2 main rooms (B); an overnight shelter housing up to 100 men in 2 main rooms (C). Shelters have onsite indoor bathrooms with sinks and soap. Residents from shelters B and C used shelter A’s day centre services.	Low – the estimated effect may be substantially different to the true effect	Report suggested crowding, use of communal sleeping arrangements and challenges enforcing physical distancing as factors associated with transmission.
[34]	Epidemiological cross-sectional analysis of data on cases from the outbreak on the Diamond Princess cruise ship, to identify transmission risk factors	Low – the estimated effect may be substantially different to the true effect	After 6 February, when passengers were confined to their cabins, passenger transmission was limited to close contacts (sharing a cabin). The absence of any cross-room transmission among passengers after the quarantine period began supports the hypothesis that transmission was via droplets/fomites and not airborne via the air conditioning system.

The four studies we found which report on transmission in communal contexts are consistent with the conveyance of risk through close contact daily living activities. It is striking that the SAR reported in the care home [71] is an order of magnitude higher than that reported in the senior assisted and independent living community [72], a much less communal setting, where elderly residents lived largely independently in separate apartments. It is important to note, however, that although the age profile in the two settings is likely to be similar, the residents of the nursing home were likely frailer. Also, ascertainment of the denominator in the care home study was not precise, so these results are uncertain.

### What evidence is there for the appropriate length of distancing between people?

Our findings are consistent with the hypothesis that the main route of CoV-2 transmission is through person-to-person short-range transmission, which occurs through large respiratory droplets ejected while speaking, coughing and sneezing. The distance that these respiratory droplets travel before falling to the ground depends on (among other factors) how they were generated. The physical behaviour of droplets is well characterised: those generated by speaking fall to the ground within 1 m or closer to the speaker [38]; droplets generated by coughing travel about 2 m [17] and those generated by sneezing can travel 8 m before falling to the ground [36]. On the basis of this evidence, our review finds no evidence to support a relaxation of the 2 m social distancing recommendation (**Table 13**).

**Table 13:** Evidence for the appropriate length of physical distancing

Reference	Study type	Overall study quality from critical appraisal	Relevant results
What evidence is there for the appropriate length of distancing between people? The evidence for this question comes from experimental and analytical studies of varying quality. The evidence is consistent with maintaining current physical distancing recommendations of 2 m.			
[36]	Non-systematic review of physics of turbulent gas clouds and implications for SARS-CoV-2 transmission	Low – the estimated effect may be substantially different to the true effect	Traditional dichotomised models, which characterise particles as either large droplets or small aerosols are over-simplified. Recent research suggests that coughing etc. emits a turbulent gas cloud consisting of a continuum of droplet sizes, which extends further than 1-2 m. This has implications for physical distancing recommendations.
[17]	Experimental fluid mechanics study – physical properties and behaviour of different particle sizes	High – very confident that the estimated effect is close to the true effect	Larger droplets (diameters of the order of 100-1000 μm) follow a ballistic trajectory (ie, they fall mostly under the influence of gravity) and reach the ground within a few seconds and without time to evaporate. The distance they travel before landing depends on (among other factors) how they were generated: those generated by coughing travel about 2 m before falling to the ground. Small droplets (aerosols) behave differently. Because they are small, they fall so slowly through the air that they have time to evaporate and can then remain suspended in the air for long periods. Aerosolised particles are ejected in a jet-like flux which, within a few metres, increases in diameter from a few centimetres to tens of centimetres. This flux bends upwards because it is warmer than the surrounding air. These particles can thus travel long distances on air flows before eventually landing.
[38]	Analytical study which proposes a simple physical model for the evaporation and movement of droplets expelled during respiratory activities	Moderate – the estimated effect is likely to be close to the true effect but there is a possibility that it is substantially different	Study suggests that the largest droplets that would completely evaporate before falling 2 m away are between 60 and 100 microns, and these expelled large droplets are carried more than 6 m away by exhaled air at a velocity of 50 m/s (sneezing), more than 2 m away at a velocity of 10 m/s (coughing) and less than 1 m away at a velocity of 1 m/s (breathing).

## DISCUSSION

This rapid evidence review integrates evidence from epidemiological, microbiological and fluid mechanics perspectives on the transmission of covid-19 in indoor settings. We found epidemiological, mechanical and microbiological evidence consistent with person-to-person, short-range spread via mostly respiratory droplets that directly reach recipients either through the air or through touching contaminated surfaces and then transferring the virus on the hands to mucosal membranes. Evidence from numerical simulation and fluid mechanics studies, microbiological laboratory studies and environmental sampling studies suggest that aerosol transmission is theoretically possible and is another potential source of transmission but we did not find conclusive epidemiological evidence to confirm this. However, evidence from fluid mechanics experiments and numerical simulations indicate that ventilation can play an important role in reducing disease transmission through diluting and dispersing the concentrations of viral particles in the air. Although viral RNA can be detected in faeces of affected individuals, we found no evidence for the presence of live virus in faecal samples nor for transmission through infected faeces.

Evidence from household, communal residential, community and workplace settings suggests that close and prolonged physical contact is important in transmission dynamics. Within households, the risk of transmission was higher between spouses than between other types of relative. Community and social settings associated with a higher risk of transmission are also those where people gather in close proximity indoors for prolonged periods. Churches and religious gatherings, sharing meals and bathing facilities, close physical contact and activities such as singing together have all been reported in conjunction with outbreaks. In contrast, there have been fewer reports of transmission in relation to more casual, short term social contact, although this may be because such contacts are subject to recall bias and harder to track and trace. Many of the workplace settings where outbreaks have occurred are characterised by close physical contact and prolonged time spent in crowded indoor spaces. Evidence from the study on outbreaks in meat and poultry processing plants [74] also highlights the role health inequalities and inadequate social protection play in relation to people continuing to work whilst ill, overcrowded housing and transportation to and from work and inadequate health and safety communication and training, particularly for non-English speakers and non-literate workers.

To our knowledge, this is the only review focusing on indoor transmission across different indoor contexts and combining evidence from epidemiological, microbiological and fluid mechanics studies. The key advantage of bringing together evidence from different disciplines in this way is that it enables practical issues that are of direct and immediate importance to decision-makers to be addressed. Three recently published systematic reviews address similar questions: Koh and colleagues estimated a pooled secondary attack rate in household settings of 18.1% (95% CI = 15.7, 20.6) – somewhat higher than our estimate, potentially reflecting the small number and poor quality of the primary studies we found on this topic. [2]. Consistent with our findings, this review also found that household transmission rates were highest between spouses. A review on clusters of SARS-CoV-2 infections highlighted the importance of disease clusters in driving transmission [3]. This study reported on disease clusters in families, communities, health care settings, religious and other gatherings, workplaces, conferences and shopping malls, again consistent with our findings. Finally, Chu and colleagues conducted a systematic review of observational epidemiological studies in order to estimate safe physical distancing [1], estimating a pooled adjusted odds ratio of 0.18 (95% confidence interval 0.09, 0.38) with physical distancing of 1 m or more, compared with a distance of less than 1 m.

Our findings have several implications for researchers, policymakers and the general public. First, we highlight important gaps in the evidence base: although aerosolised transmission is theoretically possible, whether this actually occurs in non-clinical indoor settings remains uncertain and many questions remain unanswered. It is still not known what quantity of live virus is required to present an infection risk or whether live virus is present in sufficient quantities in aerosolised particles to present a risk. Further research on these questions is urgently warranted. Although we excluded evidence from animal studies in this review, such studies should be included in future reviews, as they can potentially provide direct experimental evidence on airborne transmission [81]. Second, although there is currently no evidence for faecal-oral transmission of SARS-CoV-2, given the demonstrable potential for viral transmission via defective plumbing systems as shown in the SARS-CoV pandemic of 2003, ongoing surveillance of the potential for faecal-oral transmission would be prudent. Third, evidence from laboratory studies investigating the persistence of infectious virus on surfaces underline the ongoing importance of assiduous hand hygiene, although the precise contribution of fomite vs droplet transmission remains unclear. Finally, evidence from fluid mechanics experiments and numerical simulations reinforce the importance of maintaining the recommended physical distance and of ventilating indoor spaces to reduce the risk of transmission.

This review has a number of limitations. Although the focus of this study is transmission of SARS-CoV-2 in indoor, non-clinical settings, most of the microbiological and environmental evidence was generated in clinical contexts because this is where most of this type of study have been conducted to date. Clearly such settings are very different from non-clinical, community contexts: for example, there is a higher risk of transmission via aerosol generating procedures (AGP) and greater numbers of individuals infected with SARS-CoV-2, so virus detection in these settings is likely higher than in non-clinical indoor settings. To maximise the transferability and generalisability of these findings to community settings, we attempted to extract and report only on samples taken from areas of hospitals accessible to visitors and the general public; however, this was not always possible, as the studies did not provide information on the extent to which AGPs were carried out in patient rooms. Therefore, these results must be treated with caution in applying them to non-clinical settings.

The quality of the available epidemiological evidence was poor, so this makes any conclusions uncertain. In particular, there is significant variability in contact tracing approaches across different countries and even different regions within countries. Contact tracing of rapidly evolving infectious diseases inevitably contains case ascertainment biases, non-homogenous sampling over time and location, and uncontrolled correlation [82]. There may be publication bias, with large outbreaks potentially more likely to be reported and investigated than household studies. This review draws on evidence from a wide variety of populations and so not all the results will be directly applicable to a given population. Finally, this review was conducted at particular stage of the pandemic and as such is a snapshot in time: social contexts and drivers of behaviour and transmission will likely evolve and change as the pandemic progresses. In particular, the recent emergence of a variant of concern in the UK (VOC-202012/01) which is substantially more transmissible than other variants [83] warrants further investigation to understand transmission dynamics.

## Additional material

Online Supplementary Document
