# Supplementary material for: Which factors influence the extent of indoor transmission of SARS-CoV-2? A rapid evidence review
Source: J Glob Health. 2021 Apr 3;11:10002. doi: 10.7189/jogh.11.10002 (PMC8021073; doi:10.7189/jogh.11.10002)
Supplement: Online Supplementary Document [file jogh-11-10002-s001.pdf]

# Appendix S1 - Database search histories

**Databases/sources**

| Epidemiology and microbiological searches | Mechanics of indoor transmission searches |
|---|---|
| PubMed<br>medRxiv | PubMed<br>medRxiv<br>arXiv<br>Scopus<br>WHO COVID-19 database<br>Compendex & Inspec |

**Epidemiology and microbiological searches**

**PubMed**

Date of search 20200520

875 results

*(("Betacoronavirus"[Mesh] OR "Coronavirus Infections"[MH] OR "Spike Glycoprotein, COVID-19 Virus"[NM] OR "COVID-19"[NM] OR "Coronavirus"[MH] OR "Severe Acute Respiratory Syndrome Coronavirus 2"[NM] OR 2019nCoV[ALL] OR Betacoronavirus*[ALL] OR Corona Virus*[ALL] OR Coronavirus*[ALL] OR Coronovirus*[ALL] OR CoV[ALL] OR CoV2[ALL] OR COVID[ALL] OR COVID19[ALL] OR COVID-19[ALL] OR HCoV-19[ALL] OR nCoV[ALL] OR "SARS CoV 2"[ALL] OR SARS2[ALL] OR SARSCoV[ALL] OR SARS-CoV[ALL] OR SARS-CoV-2[ALL] OR Severe Acute Respiratory Syndrome CoV*[ALL]) AND ((2019/11/17[EDAT] : 3000[EDAT]) OR (2019/11/17[PDAT] : 3000[PDAT])))*

*AND*

*(("Disease Transmission, Infectious"[Mesh] OR "transmission" [Subheading] OR "Infections"[Mesh:NoExp] OR "Carrier State"[Mesh] OR "transmission"[Text Word] OR "transmissibility"[Text Word] OR infecti*[Text Word] OR contagi*[Text Word] OR outbreak*[Text Word] OR spread*[Text Word] OR "carrier*"[Text Word] OR "cluster"[Text Word] OR "clusters"[Text Word] OR "serial interval"[Text Word] OR "cases"[Text Word])*

*AND*

*"indoor*"[Text Word] OR "public space*"[Text Word] OR "public transport*"[Text Word] OR "closed facilit*"[Text Word] OR "public facilities"[Text Word] OR "shop*"[Text Word] OR "mall*"[Text Word] OR "shopping centre"[Text Word] OR "shopping center"[Text Word] OR "retail park"[Text Word] OR "restaurant*"[Text Word] OR "eatery"[Text Word] OR "eateries"[Text Word] OR "cafe*"[Text Word] OR "canteen"[Text Word] OR "refectory"[Text Word] OR "bar"[Text Word] OR bars OR "pub"[Text Word] OR "pubs"[Text Word] OR "nightclub*"[Text Word] OR "night-club*"[Text Word] OR "cinema*"[Text Word] OR "theatre*"[Text Word] OR "choir*"[Text Word] OR "museums"[Text Word] OR "gym*"[Text Word] OR "leisure centre"[Text Word] OR "leisure center*"[Text Word] OR "sports centre"[Text Word] OR "sports center"[Text Word] OR "workplace"[Text Word] OR "desk"[Text Word] OR "factory"[Text Word] OR "factories"[Text Word] OR "office*"[Text Word] OR "library"[Text Word]*

*OR "libraries"[Text Word] OR "multiple occupancy"[Text Word] OR "residential"[Text Word] OR (("accommodation"[Text Word] OR "residence"[Text Word]) AND ("temporary"[Text Word] OR "student"[Text Word] OR "living"[Text Word] OR "breakfast"[Text Word] OR "hostel"[Text Word] OR "rental"[Text Word])) OR (("housing"[Text Word] OR "flat"[Text Word]) AND ("tower"[Text Word] OR "block"[Text Word])) OR "multiple tenancy"[Text Word] OR "House in multiple occupation"[Text Word] OR "hotel"[Text Word] OR "prison*"[Text Word] OR "shelter"[Text Word] OR "asylum"[Text Word] OR "refugee camp"[Text Word] OR "care home"[Text Word] OR "residential home"[Text Word] OR "nursing home"[Text Word] OR "washroom"[Text Word] OR "light switch*"[Text Word] OR "door"[Text Word] OR "door handle"[Text Word] OR "toilet"[Text Word] OR "bathroom"[Text Word] OR "sink"[Text Word] OR "tap"[Text Word] OR "elevator"[Text Word] OR "lift"[Text Word] OR "escalator"[Text Word] OR "railing"[Text Word] OR "plastic"[Text Word] OR "glass"[Text Word] OR "metal"[Text Word] OR "surface"[Text Word] OR "public transport"[Text Word] OR "transport*"[Text Word] OR "car"[Text Word] OR "bus"[Text Word] OR "plane"[Text Word] OR "aeroplane*"[Text Word] OR "airplane*"[Text Word] OR "airport*"[Text Word] OR "ship"[Text Word] OR "boat*"[Text Word] OR "cruise*"[Text Word] OR "taxi*"[Text Word] OR "train"[Text Word] OR "trains"[Text Word] OR "station"[Text Word] OR "subway"[Text Word] OR ("tube"[Text Word] AND "underground"[Text Word]) OR "church"[Text Word] OR "mosque"[Text Word] OR "synagogue"[Text Word] OR "chapel"[Text Word] OR "temple"[Text Word] OR "religious gather*"[Text Word] OR "clinic"[Text Word] OR ("hospital"[Text Word] AND ("ward"[Text Word] OR "room"[Text Word]))*

**MedRxiv via** https://mcguinlu.shinyapps.io/medrxivr/

Date of search 20200521

Date range: from entry date 20191117-date of search

82 results

*Topic clusters below combined internally with OR and between clusters with AND*

*Covid cluster*

COVID-19

[Cc]oronavirus

SARS-CoV-2

2019-nCoV

*Transmission terms cluster combined with OR:*

[Cc]ontagi

[Oo]utbreak

\\b [Ss]pread

[Tt]ransmiss

[Ii]nfect

[Cc]luster

[Vv]iral load

[Cc]arrier

[Cc]ase

[Ss]hedding

*Location cluster*

[Ii]ndoor

[Ii]nside

[Ff]acilit

[Pp]ublic transport

[Rr]estaurant

[Ww]orkplace

[Pp]rison

[Ss]helter

[Cc]amp

[Tt]oilet

[Bb]athroom

[Aa]irport

[Aa]irplane

[Cc]ruise

[Rr]eligious

[Oo]bjects

[Dd]oorknob

[Hh]ousehold

**Mechanics of indoor transmission searches**

**PubMed**

Date of search 20200521

420 results

(("Severe Acute Respiratory Syndrome"[MeSH Terms] OR "sars virus"[MeSH Terms] OR "SARS"[Text Word] OR "SARS-CoV"[Text Word] OR "Severe Acute Respiratory Syndrome"[Text Word] OR "respiratory virus"[Text Word] OR "respiratory tract infection"[Text Word] OR "Respiratory Syncytial Virus"[Text Word] OR "RSV"[Text Word] OR (("Betacoronavirus"[Mesh] OR "Coronavirus Infections"[MH] OR "Spike Glycoprotein, COVID-19 Virus"[NM] OR "COVID-19"[NM] OR "Coronavirus"[MH] OR "Severe Acute Respiratory Syndrome Coronavirus 2"[NM] OR 2019nCoV[ALL] OR Betacoronavirus*[ALL] OR Corona Virus*[ALL] OR Coronavirus*[ALL] OR Coronovirus*[ALL] OR CoV[ALL] OR CoV2[ALL] OR COVID[ALL] OR COVID19[ALL] OR COVID-19[ALL] OR HCoV-19[ALL] OR nCoV[ALL] OR "SARS CoV 2"[ALL] OR SARS2[ALL] OR SARSCoV[ALL] OR SARS-CoV[ALL] OR SARS-CoV-2[ALL] OR Severe Acute Respiratory Syndrome CoV*[ALL]) AND ((2019/11/17[EDAT] : 3000[EDAT]) OR (2019/11/17[PDAT] : 3000[PDAT])))) AND ("environment, controlled"[MeSH Terms] OR "air conditioning"[MeSH Terms] OR "ventilation"[MeSH Terms] OR "sanitary engineering"[MeSH Terms] OR "air condition*"[Title/Abstract] OR "building ventilation"[Title/Abstract] OR "ventilation system"[Title/Abstract] OR "indoor ventilation"[Title/Abstract] OR "plumbing"[Title/Abstract] OR "water supply"[Title/Abstract] OR "sewage"[Title/Abstract] OR "waste water"[Title/Abstract] OR "Turbulent Flows"[Title/Abstract] OR "turbulent jets"[Title/Abstract] OR "air–mucus interaction"[Title/Abstract] OR "flow-induced"[Title/Abstract] OR "flow phenomena"[Title/Abstract] OR "Flow physics"[Title/Abstract])) AND ("Disease Transmission, Infectious"[Mesh] OR "transmission" [Subheading] OR "Infections"[Mesh:NoExp] OR transmission[Text Word] OR transmissibility[Text Word] OR infect*[Text Word] OR contagi*[Text Word] OR outbreak*[Text Word] OR spread*[Text Word])

**medRxiv** via https://mcguinlu.shinyapps.io/medrxivr/

Date of search: 20200520

9 results

*Viral respiratory terms cluster combined with OR:*

COVID-19

[Cc]oronavirus

\\bSARS\\b

2019-nCoV

[Ss]evere [Aa]cute [Rr]espiratory [Ss]yndrome

[Vv]irus

[Aa]irborne disease

*Building air etc. terms cluster combined with OR:*

[Aa]ir condition

[Bb]uilding ventilation

[Vv]entilation system

[Ii]ndoor ventilation

[Pp]lumbing

[Ww]ater supply

[Ss]ewage

[Ww]aste water

[Tt]urbulent flows

[Tt]urbulent jets

[Aa]ir–mucus interaction

[Ff]low-induced

[Ff]low phenomena

[Ff]low physics

*Transmission terms cluster combined with OR:*

[Tt]ransmiss

[Ii]nfect

[Cc]ontagi

[Oo]utbreak

\\b [Ss]pread

**arXiv**

Date of search 20200520

3 results

all=Betacoronavirus* OR Coronavirus* OR "Severe Acute Respiratory Syndrome" OR 2019nCoV OR "Corona Virus" OR Coronavirus* OR Coronovirus* OR CoV OR CoV2 OR COVID OR COVID19 OR COVID-19 OR HCoV-19 OR nCoV OR "SARS CoV 2" OR SARS2 OR SARSCoV OR SARS-CoV OR SARS-CoV-2

AND

all="air conditioned" OR "air conditioning" OR "flow physics" OR "flow phenomena" OR "building ventilation" OR "ventilation system" OR "indoor ventilation" OR sewage OR wastewater OR "waste water" OR plumbing OR "water supply"

**WHO COVID-19 database** https://search.bvsalud.org/global-literature-on-novel-coronavirus-2019-ncov/

Date of search 20200520

45 results

"air conditioned" OR "air conditioning" OR "flow physics" OR "flow phenomena" OR "building ventilation" OR "ventilation system" OR "indoor ventilation" OR sewage OR wastewater OR "waste water" OR plumbing OR "water supply"

**SCOPUS**

Date of search 20200520

182 results

Betacoronavirus* OR Coronavirus* OR "Severe Acute Respiratory Syndrome” OR 2019nCoV OR “Corona Virus*” OR Coronavirus* OR Coronovirus* OR CoV OR CoV2 OR COVID OR COVID19 OR COVID-19 OR HCoV-19 OR nCoV OR "SARS CoV 2" OR SARS2 OR SARSCoV OR SARS-CoV OR SARS-CoV-2

AND

"air conditioned" OR "air conditioning" OR "flow physics" OR "flow phenomena" OR "building ventilation" OR "ventilation system" OR "indoor ventilation" OR sewage OR wastewater OR "waste water" OR plumbing OR "water supply"

AND

transmission OR transmissibility OR infect* OR contagi* OR outbreak* OR spread*

**Compendex & Inspec**

Date of search 20200521

73 results (incl duplicates between these two databases)

(((Betacoronavirus* OR Coronavirus* OR "Severe Acute Respiratory Syndrome" OR 2019nCoV OR "Corona Virus*" OR Coronavirus* OR Coronovirus* OR CoV OR CoV2 OR COVID OR COVID19 OR COVID-19 OR HCoV-19 OR nCoV OR "SARS CoV 2" OR SARS2 OR SARSCoV OR SARS-CoV OR SARS-CoV-2) WN ALL) AND (("air conditioned" OR "air conditioning" OR "flow physics" OR "flow phenomena" OR "building ventilation" OR "ventilation system" OR "indoor ventilation" OR sewage OR wastewater OR "waste water" OR plumbing OR "water supply") WN ALL))

# Appendix S2 – Critical appraisal checklists

## Critical Appraisal Checklist – Laboratory Studies

Adapted from: Public Health Agency of Canada, Infection Prevention and Control Guidelines, Critical Appraisal Toolkit: http://publications.gc.ca/collections/collection_2014/aspc-phac/HP40-119-2014-eng.pdf & http://syrf.org.uk/protocols/ & https://casp-uk.net/wp-content/uploads/2018/01/CASP-Diagnostic-Checklist-2018.pdf

1. **Research question**
   Is there a clear question for the study to address?

2. **Methodology**
   - Are all experimental procedures presented clearly and in sufficient detail in the methods section or appendix?
   - Does the paper describe how many times experiments are repeated?
   - Evidence of experimental controls for validity or an appropriate reference standard?
   - Tools/experiments are known and shown to be valid and reliable?

3. **Assessment for control of analysis**
   - Are summary estimates defined as median or mean?
   - Are all data presented as figures? (including error bars)
   - Is it clear if error bars refer to standard deviation (SD), confidence interval (CI) or standard error of the mean (SEM)?
   - Is it clear which statistical tests have been used?
   - Are statistical tests appropriate for level of data and hypothesis being tests?

4. **Ethics (if applicable)**
   - Study was approved by appropriate ethics review board or sufficient details that conduct was ethical.

5. **Applicability**
   Are the results from this study generalisable and applicable to non-laboratory settings?

## Critical Appraisal Checklist – Contact Tracing Studies

Adapted from Joanna Briggs Institute checklist for evaluating case series: critical-appraisal-tools - Critical Appraisal Tools | Joanna Briggs Institute

- Were there clear criteria for inclusion in the case series? Was a case/contact definition provided?
- Was the index case clearly identified/described?
- Was the condition measured in a standard, reliable way for all participants included in the case series?
- Were valid methods used for identification of the condition for all participants included in the case series?
- Did the case series have consecutive inclusion of participants?
- Did the case series have complete inclusion of participants?
- Was there clear reporting of the demographics of the participants in the study?
- Was there clear reporting of clinical information of the participants?
- Was there a surveillance/monitoring period for all identified cases/contacts? (other than clinical monitoring of condition)
- Were the outcomes or follow up results of cases clearly reported? ie secondary cases &tertiary cases
- Were nonsypmtomatic patients tested in the same way as symptomatic patients?
- Was there a follow up test within a week of first test? (for non-symptomatic)
- Was statistical analysis appropriate?

## Critical Appraisal Checklists – Numerical Simulation Studies

Adapted from:

American Society of Mechanical Engineers. (2009). Standard for Verification and Validation in Computational Fluid Dynamics and Heat Transfer (Vol. VV 20): American Society of Mechanical Engineers.

American Society of Mechanical Engineers. (2018). Assessing Credibility of Computational Modeling through Verification and Validation: Application to Medical Devices (Vol. VV 40): American Society of Mechanical Engineers.

Roache, P. J. (2009). Fundamentals of verification and validation: hermosa publ.

- What was the model designed to simulate?
- What were the author's conclusions?
- Is the programming and computational implementation of the conceptual model correct?
- Was an uncertainty analysis undertaken?
- Was the model validated at the level of the computed uncertainty?
- Can the skillset of the study team be evaluated in reference to the performance requirements for the simulations being undertaken?
- Are the physical phenomena governing the physical reality modelled?
- Are the conclusions justifiable by the nature of the reality being described?
- What were the study's methodological limitations?
- What were the study's limitations in generalisability to the real world?
- Did the authors make any suggestions for future research?
- Strength of evidence

## Appendix S3: List of articles retained for analysis – description, quality assessment, topic questions

| Reference | Study description | Study quality | 1 – Aerosol transmission | 2 - Faecal-oral | 3 - Ventilation systems | 4 - Plumbing systems | 5 - Fomite transmission | 6 - Residential settings | 7 - Workplaces | 8 - Other indoor settings | 9 - Activities | 10 – Physical distancing |
|---|---|---|---|---|---|---|---|---|---|---|---|---|
| Asadi S, Wexler AS, Cappa CD, Barreda S, & Bouvier NM, e. a. (2020). Effect of voicing and articulation manner on aerosol particle emission during human speech. PLOS ONE 15, e0227699. Retrieved from https://doi.org/10.1371/journal.pone.0227699 | Experimental study measuring the emission rate of respiratory aerosols in human subjects when voicing different sounds, both in normal speech and as isolated sounds. | High - very confident that the estimated effect is close to the true effect | | | | | | | | | x | |
| Asadi, S., Wexler, A. S., & Cappa, C. D. e. a. (2019). Aerosol emission and superemission during human speech increase with voice loudness. Sci Rep, 9. Retrieved from https://doi.org/10.1038/s41598-019-38808-z | Experimental study in which human subjects repeatedly said the vowel sound in the word "saw" but at different amplitudes. The volume of particles emitted was measured. | High - very confident that the estimated effect is close to the true effect | | | | | | | | | x | |
| Bi, Q., Wu, Y., Mei, S., Ye, C., Zou, X., Zhang, Z., . . . Feng, T. (2020). Epidemiology and Transmission of COVID-19 in Shenzhen China: Analysis of 391 cases and 1,286 of their close contacts. doi:10.1101/2020.03.03.20028423 | Epidemiological analysis of symptomatic surveillance and contact tracing data for 391 SARS-CoV-2 cases and 1286 controls identified from 14 January – 12 February 2020, Shenzhen, China. Purpose of study was to estimate metrics of transmission and investigate transmission risk factors. The researchers followed up cases and close contacts for 14 days and then retested. Close contacts were defined as people living in the same apartment, sharing a meal, travelling together, or interacting socially with the index case from 2 days before the onset of symptoms. | Low - the estimated effect may be substantially different to the true effect | | | | | | x | | | x | |
| Bourouiba, L. (2020). Turbulent Gas Clouds and Respiratory Pathogen Emissions: Potential Implications for Reducing Transmission of COVID-19. Jama. doi:10.1001/jama.2020.4756 | Non-systematic review of physics of turbulent gas clouds and implications for SARS-CoV-2 transmission | Low - the estimated effect may be substantially | x | | | | | | | | | x |

| Reference | Study description | Study quality | 1 – Aerosol transmission | 2 - Faecal-oral | 3 - Ventilation systems | 4 - Plumbing systems | 5 - Fomite transmission | 6 - Residential settings | 7 - Workplaces | 8 - Other indoor settings | 9 - Activities | 10 – Physical distancing |
|---|---|---|---|---|---|---|---|---|---|---|---|---|
| | | different to the true effect | | | | | | | | | | |
| Bourouiba, L., Dehandschoewercker, E., & Bush, J. (2014). Violent expiratory events: On coughing and sneezing. Journal of Fluid Mechanics, 745, 537-563. doi:10.1017/jfm.2014.88 | Experimental fluid mechanics study - physical properties and behaviour of different particle sizes | High - very confident that the estimated effect is close to the true effect | x | | | | | | | | | x |
| Burke, R. M., Balter, S., Barnes, E., Barry, V., Bartlett, K., Beer, K. D., . . . Hunte. (2020). Enhanced Contact Investigations for Nine Early Travel-Related Cases of SARS-CoV-2 in the United States. doi:10.1101/2020.04.27.20081901 | Epidemiological contact tracing study of the first 9 travel-related cases identified in the USA, and 338 of their close contacts - follow up of close contacts to identify transmission risk factors. | Very low - the estimated effect is very uncertain | | | | | | x | | x | x | |
| Chao, C., Wan, M. P., Morawska, L., Johnson, G. R., Ristovski, Z. D., Hargreaves, M., . . . Katoshevski, D. (2009). Characterization of expiration air jets and droplet size distributions immediately at the mouth opening. Journal of aerosol science, 40, 122–133 | Laboratory experimental study which measured expired droplets from human subjects coughing and speaking (counting from 1 to 100). Expiration velocities and droplet size distributions were measured. | High - very confident that the estimated effect is close to the true effect | | | | | | | | | x | |
| Chaw, L., Koh, W. C., Jamaludin, S. A., Naing, L., Alikhan, M. F., & Wong, J. (2020). SARS-CoV-2 transmission in different settings: Analysis of cases and close contacts from the Tablighi cluster in Brunei Darussalam. doi:10.1101/2020.05.04.20090043 | Epidemiological analysis of contact tracing data linked to an outbreak centred on an Islamic religious gathering (Tablighi Jama'at) in Kuala Lumpur, Malaysia and attended by 75 citizens of Brunei, of whom 19 became ill. There were a further 52 additional secondary/subsequent cases in Brunei, bringing the cluster size to 71. Study investigates environmental, behavioural and host risk factors for transmission. The study also investigated attack rates for different relationships living together in households | Low - the estimated effect may be substantially different to the true effect | | | | | | x | | x | x | |
| Chen, C., Zhao, B., & Yang, X. (2011). Significance of two-way airflow effect due to temperature difference in indoor air quality. | Experimental case studies modelling the two-way airflow effect due to temperature difference in indoor air quality | High - very confident that the estimated effect | | | x | | | | | | | |

| Reference | Study description | Study quality | 1 – Aerosol transmission | 2 - Faecal-oral | 3 - Ventilation systems | 4 - Plumbing systems | 5 - Fomite transmission | 6 - Residential settings | 7 - Workplaces | 8 - Other indoor settings | 9 - Activities | 10 – Physical distancing |
|---|---|---|---|---|---|---|---|---|---|---|---|---|
| | | is close to the true effect | | | | | | | | | | |
| Cheng, H. Y., Jian, S. W., Liu, D. P., Ng, T. C., Huang, W. T., Taiwan Covid-19 Outbreak Investigation, T., & Lin, H. H. (2020). High transmissibility of COVID-19 near symptom onset. doi:10.1101/2020.03.18.20034561 | Epidemiological analysis of contact tracing data to understand transmission dynamics and estimate the infectious period. | Very low - the estimated effect is very uncertain | | | | | | x | | | x | |
| Cheng, V. C. C., Wong, S. C., Chen, J. H. K., Yip, C. C. Y., Chuang, V. W. M., Tsang, O. T. Y., . . . Yuen, K. Y. (2020). Escalating infection control response to the rapidly evolving epidemiology of the coronavirus disease 2019 (COVID-19) due to SARS-CoV-2 in Hong Kong. Infect Control Hosp Epidemiol, 41(5), 493-498. doi:10.1017/ice.2020.58. Epub 2020 Mar 5. | Description of infection control measures undertaken during the early stages of the covid-19 pandemic in Hong Kong. Study used real-time PCR methods for detection of SARS-CoV-2 from an air sample. Study also quantified the amount of virus present by reporting viral load/gene copy data. | Low - the estimated effect may be substantially different to the true effect | | | | | x | | | | | |
| Chin, A. W. H., Chu, J. T. S., Perera, M. R. A., Hui, K. P. Y., Yen, H.-L., Chan, M. C. W., . . . Poon, L. L. M. (2020). Stability of SARS-CoV-2 in different environmental conditions. The Lancet Microbe Retrieved from https://doi.org/10.1016/S2666-5247(20)30003-3 | Laboratory based study investigating the stability of SARS-CoV-2 in different environmental conditions. | Low - the estimated effect may be substantially different to the true effect | | | | | x | | | | | |
| Dyal, J. W., Grant, M. P., Broadwater, K., Bjork, A., Waltenburg, M. A., Gibbins, J. D., . . . Honein, M. A. (2020). COVID-19 Among Workers in Meat and Poultry Processing Facilities - 19 States, April 2020. MMWR Morb Mortal Wkly Rep, 69(18). doi:10.15585/mmwr.mm6918e3 | Centers for Disease Control (CDC) report on workplace outbreaks in meat and poultry processing facilities across the USA. The article presents data from 17 of 23 US states reporting at least one such outbreak, expressing the number of cases in each state as a proportion of all meat and poultry workers employed in the state. | Low - the estimated effect may be substantially different to the true effect | | | | | | | x | | | |
| Gormley, M., Aspray, T. J., Kelly, D. A., & Rodriguez-Gil, C. (2017). Pathogen cross-transmission via building sanitary plumbing systems in a full scale | Real-scale experiment investigating the role of sanitary plumbing systems in the transmission of aerosolised viruses | High - very confident that the estimated effect | | | | x | | | | | | |

| Reference | Study description | Study quality | 1 – Aerosol transmission | 2 - Faecal-oral | 3 - Ventilation systems | 4 - Plumbing systems | 5 - Fomite transmission | 6 - Residential settings | 7 - Workplaces | 8 - Other indoor settings | 9 - Activities | 10 – Physical distancing |
|---|---|---|---|---|---|---|---|---|---|---|---|---|
| pilot test-rig. PLoS One, 12(2), e0171556. doi:10.1371/journal.pone.0171556 | | is close to the true effect | | | | | | | | | | |
| Guo, Z. D., Wang, Z. Y., Zhang, S. F., Li, X., Li, L., Li, C., . . . Chen, W. (2020). Aerosol and Surface Distribution of Severe Acute Respiratory Syndrome Coronavirus 2 in Hospital Wards, Wuhan, China, 2020. Emerg Infect Dis, 26(7). doi:10.3201/eid2607.200885 | Hospital-based study in Wuhan, China which tested surface and air samples for presence of SARS-CoV-2 RNA | Moderate - the estimated effect is likely to be close to the true effect but there is a possibility that it is substantially different | x | | | | | | | | | |
| Hamner, L., Dubbel, P., Capron, I., Ross, A., Jordan, A., Lee, J., . . . Leibrand, H. (2020). High SARS-CoV-2 Attack Rate Following Exposure at a Choir Practice - Skagit County, Washington, March 2020. MMWR Morb Mortal Wkly Rep, 69(19), 606-610. doi:10.15585/mmwr.mm6919e610.15585/mmwr.mm6919e6. | Epidemiological analysis of a disease cluster linked to a choir practice in Washington State, USA | Low - the estimated effect may be substantially different to the true effect | x | | | | | | | | | |
| Hirotsu, Y., Maejima, M., Nakajima, M., Mochizuki, H., & Omata, M. (2020). Environmental cleaning is effective for the eradication of severe acute respiratory syndrome coronavirus 2 (SARS-CoV-2) in contaminated hospital rooms: A patient from the Diamond Princess cruise ship. Infect Control Hosp Epidemiol, 1-8. doi:10.1017/ice.2020.144 | Letter to the editor describing the effectiveness of hospital environmental cleaning procedures in preventing transmission from an infected case, Japan. Study used real-time PCR methods for detection of SARS-CoV-2 on 15 environmental samples from rooms occupied by an infected patient, with samples collected after thorough cleaning of the area. | Low - the estimated effect may be substantially different to the true effect | | | | | x | | | | | |
| Holshue, M. L., DeBolt, C., Lindquist, S., Lofy, K. H., Wiesman, J., Bruce, H., . . . Washington State -nCo, V. C. I. T. (2020). First Case of 2019 Novel Coronavirus in the United States. N Engl J Med, 382(10), 929-936. doi:10.1056/NEJMoa2001191 | Case report – first case in USA | Low - the estimated effect may be substantially different to the true effect | | x | | | | | | | | |

| Reference | Study description | Study quality | 1 – Aerosol transmission | 2 - Faecal-oral | 3 - Ventilation systems | 4 - Plumbing systems | 5 - Fomite transmission | 6 - Residential settings | 7 - Workplaces | 8 - Other indoor settings | 9 - Activities | 10 – Physical distancing |
|---|---|---|---|---|---|---|---|---|---|---|---|---|
| Hung, H. C. K., Chan, D. W. T., Law, L. K. C., Chan, E. H. W., & Wong, E. S. W. (2006). Industrial experience and research into the causes of SARS virus transmission in a high-rise residential housing estate in Hong Kong. Building Services Engineering Research and Technology, 27(2), 91-102. doi:10.1191/0143624406bt145oa | Field study investigating foul air and water backflow in a real-scale drainage system | High - very confident that the estimated effect is close to the true effect | | | | x | | | | | | |
| Jack, L. B., Cheng, C., & Lu, W. H. (2006). Numerical simulation of pressure and airflow response of building drainage ventilation systems. Building Services Engineering Research and Technology, 27(2), 141-152. doi:10.1191/0143624406bt152oa | Methodological paper on empirical and simulation techniques for the forensic analysis of virus spread via building drainage systems | Moderate - the estimated effect is likely to be close to the true effect but there is a possibility that it is substantially different | | | | x | | | | | | |
| Jiang, F. C., Jiang, X. L., Wang, Z. G., Meng, Z. H., Shao, S. F., Anderson, B. D., & Ma, M. J. (2020). Detection of Severe Acute Respiratory Syndrome Coronavirus 2 RNA on Surfaces in Quarantine Rooms. Emerg Infect Dis, 26(9). doi:10.3201/eid2609.201435 | Study collecting surface samples to test for presence of SARS-CoV-2 RNA, using real-time PCR methods for detection of SARS-CoV-2, in 2 rooms occupied by 2 pre-symptomatic confirmed cases in a quarantine hotel, China | Low - the estimated effect may be substantially different to the true effect | | | | | x | | | | | |
| Jiehao C, Jing X, & Daojiong L, e. a. (2020). A case series of children with 2019 novel coronavirus infection: clinical and epidemiological features. . Clin. Infect. Dis. , pii: ciaa198. | Case series – ten children in Wuhan, China | Low - the estimated effect may be substantially different to the true effect | | x | | | | | | | | |
| Li Y., Huang X., & I.T., Y. (2005). Role of air distribution in SARS transmission during the largest nosocomial outbreak in Hong Kong. . Indoor Air, 15, 83-95. | Study of environmental evidence of possible airborne transmission of SARS in a hospital ward in Hong Kong in 2003, involving retrospective measurements of ventilation systems, air sampling | High - very confident that the estimated effect | x | | | | | | | | | |

| Reference | Study description | Study quality | 1 – Aerosol transmission | 2 - Faecal-oral | 3 - Ventilation systems | 4 - Plumbing systems | 5 - Fomite transmission | 6 - Residential settings | 7 - Workplaces | 8 - Other indoor settings | 9 - Activities | 10 – Physical distancing |
|---|---|---|---|---|---|---|---|---|---|---|---|---|
| | and computational fluid dynamics simulations to analyses and predict bio-aerosol dispersion in the hospital ward. | is close to the true effect | | | | | | | | | | |
| Li, L. Y., Wu, W., Chen, S., Gu, J. W., Li, X. L., Song, H. J., . . . Cai, Q. (2020). Digestive system involvement of novel coronavirus infection: Prevention and control infection from a gastroenterology perspective. J Dig Dis, 21(4), 199-204. doi:10.1111/1751-2980.12862 | Non-systematic review - relationship between COVID-19 and the digestive system | Low - the estimated effect may be substantially different to the true effect | | x | | | | | | | | |
| Li, Y., Duan, S., Yu, I. T., & Wong, T. W. (2005). Multi-zone modeling of probable SARS virus transmission by airflow between flats in Block E, Amoy Gardens. Indoor Air, 15(2), 96-111. doi:10.1111/j.1600-0668.2004.00318.x | Numerical simulation study modelling potential airborne transmission of SARS between apartments - Amoy Gardens outbreak, Hong Kong, 2003 | Moderate - the estimated effect is likely to be close to the true effect but there is a possibility that it is substantially different | x | | x | | | | | | | |
| Li, Y., Qian, H., Hang, J., Chen, X., Hong, L., Liang, P., . . . Kang, M. (2020). Evidence for probable aerosol transmission of SARS-CoV-2 in a poorly ventilated restaurant. doi:10.1101/2020.04.16.20067728 | Numerical simulation study (real-scale experiment and computational fluid dynamics simulation), demonstrating probable aerosol transmission of SARS-CoV-2, at an outbreak in a restaurant in Guangzhou, China | High - very confident that the estimated effect is close to the true effect | x | | x | | | | | | | |
| Lim, T., Cho, J., & Kim, B. S. (2011). Predictions and measurements of the stack effect on indoor airborne virus transmission in a high-rise hospital building. Build Environ, 46(12), 2413-2424. doi:10.1016/j.buildenv.2011.04.015 | Numerical simulation and field experiment investigating airborne transmission within a high rise building | Very low - the estimated effect is very uncertain | | | | x | | | | | | |
| Ling, Y., Xu, S. B., Lin, Y. X., Tian, D., Zhu, Z. Q., Dai, F. H., . . . Lu, H. Z. (2020). Persistence and clearance of viral RNA in 2019 novel coronavirus disease | Case series – 66 convalescent adult patients, Shanghai, China | Low - the estimated effect may be substantially | | x | | | | | | | | |

| Reference | Study description | Study quality | 1 – Aerosol transmission | 2 - Faecal-oral | 3 - Ventilation systems | 4 - Plumbing systems | 5 - Fomite transmission | 6 - Residential settings | 7 - Workplaces | 8 - Other indoor settings | 9 - Activities | 10 – Physical distancing |
|---|---|---|---|---|---|---|---|---|---|---|---|---|
| rehabilitation patients. Chin Med J (Engl), 133(9), 1039-1043. doi:10.1097/CM9.0000000000000774 | | different to the true effect | | | | | | | | | | |
| Liu, Y., Ning, Z., Chen, Y., Guo, M., Liu, Y., Gali, N. K., . . . Lan, K. (2020). Aerodynamic analysis of SARS-CoV-2 in two Wuhan hospitals. Nature. doi:10.1038/s41586-020-2271-3 | Hospital-based study to measure the concentration of SARS-CoV-2 RNA in aerosols in 2 hospitals in Wuhan, China | Moderate - the estimated effect is likely to be close to the true effect but there is a possibility that it is substantially different | x | x | | | | | | | | |
| McMichael, T. M., Clark, S., Pogosjans, S., Kay, M., Lewis, J., Baer, A., . . . Duchin, J. S. (2020). COVID-19 in a Long-Term Care Facility - King County, Washington, February 27-March 9, 2020. MMWR Morb Mortal Wkly Rep, 69(12), 339-342. doi:10.15585/mmwr.mm6912e1 | Epidemiological report on an outbreak in a residential elderly care facility in Washington State, USA (resulting in 81 residents, 34 staff members, and 14 visitors becoming ill) | Very low - the estimated effect is very uncertain | | | | | | x | x | | x | |
| Mittal, R., Ni, R., & Seo, J.-H. (2020). The flow physics of COVID-19. Journal of Fluid Mechanics, 894. doi:10.1017/jfm.2020.330 | Non-systematic review on the flow physics of COVID-19 | Low - the estimated effect may be substantially different to the true effect | x | | | | | | | | | |
| Niu, J., & Tung, T. C. (2008). On-site quantification of re-entry ratio of ventilation exhausts in multi-family residential buildings and implications. Indoor Air, 18(1), 12-26. doi:10.1111/j.1600-0668.2007.00500.x | Experimental fluid mechanics study using tracer gas to study the transmission of airborne particles around an apartment building | Moderate - the estimated effect is likely to be close to the true effect but there is a possibility that it is substantially different | | | x | | | | | | | |

| Reference | Study description | Study quality | 1 – Aerosol transmission | 2 - Faecal-oral | 3 - Ventilation systems | 4 - Plumbing systems | 5 - Fomite transmission | 6 - Residential settings | 7 - Workplaces | 8 - Other indoor settings | 9 - Activities | 10 – Physical distancing |
|---|---|---|---|---|---|---|---|---|---|---|---|---|
| Poussou, S. B., & Plesniak, M. W. (2012). Vortex dynamics and scalar transport in the wake of a bluff body driven through a steady recirculating flow. Exp Fluids, 53(3), 747-763. doi:10.1007/s00348-012-1325-1 | Experimental fluid dynamics study investigating the pattern of air flow within a commercial aircraft. | High - very confident that the estimated effect is close to the true effect | x | | | | | | | | | |
| Pung, R., Chiew, C. J., Young, B. E., Chin, S., Chen, M. I., Clapham, H. E., . . . Lee, V. J. M. (2020). Investigation of three clusters of COVID-19 in Singapore: implications for surveillance and response measures. Lancet, 395(10229), 1039-1046. doi:10.1016/S0140-6736(20)30528-6. Epub 2020 Mar 17. | Epidemiological outbreak study analysing contact tracing data on various outbreaks in Singapore. | Very low - the estimated effect is very uncertain | | | | | x | | x | x | | |
| Riddell, S., Goldie, S., Hill, A., Eagles, D., & Drew, T. W. (2020). The effect of temperature on persistence of SARS-CoV-2 on common surfaces. Virology Journal, 17(1), 145. doi:10.1186/s12985-020-01418-7 | Laboratory based study investigating the survival rates of infectious SARS-CoV-2 on common surfaces (cotton, glass, steel, vinyl, paper and polymer banknotes) at three different temperatures (20°C, 30°C, and 40°C) with no exposure to ultraviolet light (known to rapidly deactivate the virus) and humidity controlled at 50 %. | High - very confident that the estimated effect is close to the true effect | | | | | x | | | | | |
| Roxby, A. C., Greninger, A. L., Hatfield, K. M., Lynch, J. B., Dellit, T. H., James, A., . . . Neme, S. (2020). Detection of SARS-CoV-2 Among Residents and Staff Members of an Independent and Assisted Living Community for Older Adults - Seattle, Washington, 2020. MMWR Morb Mortal Wkly Rep, 69(14), 416-418. doi:10.15585/mmwr.mm6914e2 | Epidemiological report on an outbreak in an independent living facility for the elderly (sheltered housing) in Seattle, Washington State, USA (resulting in 4 residents testing positive) | Low - the estimated effect may be substantially different to the true effect | | | | | | x | x | | x | |
| Santarpia, J. L., Rivera, D. N., Herrera, V., Morwitzer, M. J., Creager, H., Santarpia, G. W., . . . Lowe, J. J. (2020). Transmission Potential of SARS-CoV-2 in Viral Shedding Observed at the University of Nebraska Medical Center. doi:10.1101/2020.03.23.20039446 | Hospital-based study collecting surface and air samples to test for presence of SARS-CoV-2 RNA, using real-time PCR methods for detection of SARS-CoV-2, Nebraska, USA. Study also quantified the amount of virus present by reporting viral load/gene | Low - the estimated effect may be substantially | x | x | | | x | | | | | |

| Reference | Study description | Study quality | 1 – Aerosol transmission | 2 - Faecal-oral | 3 - Ventilation systems | 4 - Plumbing systems | 5 - Fomite transmission | 6 - Residential settings | 7 - Workplaces | 8 - Other indoor settings | 9 - Activities | 10 – Physical distancing |
|---|---|---|---|---|---|---|---|---|---|---|---|---|
| | copy data. Study also attempted to culture live virus from environmental samples. | different to the true effect | | | | | | | | | | |
| Sun, Z., Cai, X., Gu, C., Zhang, R., Han, W., Qian, Y., . . . Qu, D. (2020). Stability of the COVID-19 virus under wet, dry and acidic conditions. . MedRxiv, 2020.04.09.20058875. Retrieved from https://doi.org/10.1101/2020.04.09.20058875 | Laboratory based study using a strain from the nasal-pharyngeal swab of a clinically confirmed COVID-19 patient in Shanghai, investigating the stability of SARS-CoV-2 in wet, dry and acidic conditions at room temperature. The researchers measured the stability of SARS-CoV-2 in wet (in 100 uL culture medium) and dry (10 uL supernatant on filter paper) environments at room temperature (22°C) each day for 7 days, as well as its stability under acidic conditions to mimic the gastric environment (pH2.2) | Low - the estimated effect may be substantially different to the true effect | | | | | x | | | | | |
| Sung, M., Jo, S., Lee, S. E., Ki, M., Choi, B. Y., & Hong, J. (2018). Airflow as a possible transmission route of middle east respiratory syndrome at an initial outbreak hospital in Korea. International Journal of Environmental Research and Public Health, 15(12). doi:10.3390/ijerph15122757 | Tracer gas experiments to investigate airflow patterns | Moderate - the estimated effect is likely to be close to the true effect but there is a possibility that it is substantially different | | | x | | | | | | | |
| Tang, A., Tong, Z. D., Wang, H. L., Dai, Y. X., Li, K. F., Liu, J. N., . . . Yan, J. B. (2020). Detection of Novel Coronavirus by RT-PCR in Stool Specimen from Asymptomatic Child, China. Emerg Infect Dis, 26(6), 1337-1339. doi:10.3201/eid2606.200301 | Case report – ten year old, asymptomatic boy, Zhoushan, China | Low - the estimated effect may be substantially different to the true effect | | x | | | | | | | | |
| Tobolowsky, F. A., Gonzales, E., Self, J. L., Rao, C. Y., Keating, R., Marx, G. E., . . . Kay, M. (2020). COVID-19 Outbreak Among Three Affiliated Homeless Service Sites - King County, Washington, 2020. MMWR Morb | Epidemiological report of an outbreak in 3 affiliated overnight and day centres for homeless people (comprising a 24-hour shelter serving up to 40 men and 10 women (A); an overnight shelter housing up to 110 men in 2 main rooms (B); an overnight shelter | Low - the estimated effect may be substantially | | | | | | x | x | | | |

| Reference | Study description | Study quality | 1 – Aerosol transmission | 2 - Faecal-oral | 3 - Ventilation systems | 4 - Plumbing systems | 5 - Fomite transmission | 6 - Residential settings | 7 - Workplaces | 8 - Other indoor settings | 9 - Activities | 10 – Physical distancing |
|---|---|---|---|---|---|---|---|---|---|---|---|---|
| Mortal Wkly Rep, 69(17), 523-526. doi:10.15585/mmwr.mm6917e2 | housing up to 100 men in 2 main rooms (C). Shelters have onsite indoor bathrooms with sinks and soap. Residents from shelters B and C used shelter A's day centre services. | different to the true effect | | | | | | | | | | |
| van Doremalen, N., Bushmaker, T., Morris, D. H., Holbrook, M. G., Gamble, A., Williamson, B. N., . . . Munster, V. J. (2020). Aerosol and Surface Stability of SARS-CoV-2 as Compared with SARS-CoV-1. N Engl J Med, 382(16), 1564-1567. doi:10.1056/NEJMc2004973. Epub 2020 Mar 17. | Laboratory-based study investigating the persistence of SARS-CoV-2 under various controlled conditions | High - very confident that the estimated effect is close to the true effect | x | | | | x | | | | | |
| Wölfel, R., Corman, V. M., Guggemos, W., Seilmaier, M., Zange, S., Muller, M. A., . . . Wendtner, C. (2020). Virological assessment of hospitalized patients with COVID-2019. Nature, 581(7809), 465-469. doi:10.1038/s41586-020-2196-x | Case series - virological assessment of nine hospitalised cases who acquired infection from the same index case, Germany | Very low - the estimated effect is very uncertain | | x | | | | | | | | |
| Wong, T. W., Lee, C. K., Tam, W., Lau, J. T., Yu, T. S., Lui, S. F., . . . Outbreak Study, G. (2004). Cluster of SARS among medical students exposed to single patient, Hong Kong. Emerg Infect Dis, 10(2), 269-276. doi:10.3201/eid1002.030452 | Retrospective cohort study of 66 medical students exposed to a SARS inpatient, Hong Kong, 2003. Sample consisted of 16 students with SARS and 50 healthy students. ( Study included because it involved inspections and measurements of ventilation system and air flow). | Low - the estimated effect may be substantially different to the true effect | x | | | | | | | | | |
| Wu Y, Guo C, & Tang L, e. a. (2020). Prolonged presence of SARS-CoV-2 viral RNA in faecal samples. . Lancet Gastroenterol Hepatol, 5, 434-435. doi:10.1016/S2468-1253(20)30083-2 | Case series - Real-time RT-PCR results of respiratory and faecal samples from hospitalised patients with COVID-19, Zhuhai, China, throughout the course of their illness and quarantine period. | Very low - the estimated effect is very uncertain | | x | | | | | | | | |
| Wu, S., Wang, Y., Jin, X., Tian, J., Liu, J., & Mao, Y. (2020). Environmental contamination by SARS-CoV-2 in a designated hospital for coronavirus disease 2019. Am J Infect Control. doi:10.1016/j.ajic.2020.05.003 | Hospital-based study collecting surface and air samples to test for presence of SARS-CoV-2 RNA, Wuhan, China | Moderate - the estimated effect is likely to be close to the true effect but there is a possibility that | x | | | | x | | | | | |

| Reference | Study description | Study quality | 1 – Aerosol transmission | 2 - Faecal-oral | 3 - Ventilation systems | 4 - Plumbing systems | 5 - Fomite transmission | 6 - Residential settings | 7 - Workplaces | 8 - Other indoor settings | 9 - Activities | 10 – Physical distancing |
|---|---|---|---|---|---|---|---|---|---|---|---|---|
| | | it is substantially different | | | | | | | | | | |
| Xie, X., Li, Y., Chwang, A. T. Y., Ho, P. L., & Seto, W. H. (2007). How far droplets can move in indoor environments – revisiting the Wells evaporation–falling curve. . Indoor Air, 17, 211-225. doi:10.1111/j.1600-0668.2007.00469.x | Analytical study which proposes a simple physical model for the evaporation and movement of droplets expelled during respiratory activities | Moderate - the estimated effect is likely to be close to the true effect but there is a possibility that it is substantially different | x | | | | | | | | | x |
| Xie, X., Li, Y., Sun, H., & Liu, L. (2009). Exhaled droplets due to talking and coughing. J. R. Soc. Interface, 6S703–S714. Retrieved from http://doi.org/10.1098/rsif.2009.0388.focus | Experiment measuring the number and size of respiratory droplets emitted during speaking and coughing. | Moderate - the estimated effect is likely to be close to the true effect but there is a possibility that it is substantially different | | | | | | | | | x | |
| Xu, P., Qian, H., Miao, T., Yen, H. L., Tan, H., Cowling, B. J., & Li, Y. J. (2020). Transmission routes of Covid-19 virus in the Diamond Princess Cruise ship. doi:10.1101/2020.04.09.20059113 | Epidemiological cross-sectional analysis of data on cases from the outbreak on the Diamond Princess cruise ship, to identify transmission risk factors | Low - the estimated effect may be substantially different to the true effect | x | | | | | x | x | | | |
| Yamagishi, T. (2020). Environmental sampling for severe acute respiratory syndrome coronavirus 2 (SARS-CoV-2) during a coronavirus disease (COVID-19) outbreak aboard a commercial cruise ship. . Retrieved from https://doi.org/10.1101/2020.05.02.20088567 | Cross-sectional study testing surface and air samples for presence of SARS-CoV-2 RNA, in cabins which were occupied by confirmed cases on the Diamond Princess cruise ship, Japan, using real-time PCR methods for detection of SARS-CoV-2. Study also quantified the amount of virus present by reporting | Low - the estimated effect may be substantially different to the true effect | x | x | | | x | | | | | |

| Reference | Study description | Study quality | 1 – Aerosol transmission | 2 - Faecal-oral | 3 - Ventilation systems | 4 - Plumbing systems | 5 - Fomite transmission | 6 - Residential settings | 7 - Workplaces | 8 - Other indoor settings | 9 - Activities | 10 – Physical distancing |
|---|---|---|---|---|---|---|---|---|---|---|---|---|
| | viral load/gene copy data. Study also attempted to culture live virus from environmental samples. | | | | | | | | | | | |
| Yang, L., Li, M., Li, X., & Tu, J. (2018). The effects of diffuser type on thermal flow and contaminant transport in high-speed train (HST) cabins–a numerical study. International Journal of Ventilation, 17(1), 48-62. doi:10.1080/14733315.2017.1351736 | Numerical simulation study investigating air flow patterns in a high speed train carriage. | Moderate - the estimated effect is likely to be close to the true effect but there is a possibility that it is substantially different | x | | | | | | | | | |
| Ye, G., Lin, H., Chen, L., Wang, S., Zeng, Z., Wang, W., . . . Wang, X. (2020). Environmental contamination of the SARS-CoV-2 in healthcare premises: An urgent call for protection for healthcare workers. doi:10.1101/2020.03.11.20034546 | Hospital-based study collecting samples from surfaces and objects to test for presence of SARS-CoV-2 RNA, using real-time PCR methods for detection of SARS-CoV-2, Wuhan, China. | Moderate - the estimated effect is likely to be close to the true effect but there is a possibility that it is substantially different | | | | | x | | | | | |
| Yu, H. C., Mui, K. W., Wong, L. T., & Chu, H. S. (2017). Ventilation of general hospital wards for mitigating infection risks of three kinds of viruses including Middle East respiratory syndrome coronavirus. Indoor and Built Environment, 26(4), 514-527. doi:10.1177/1420326X16631596 | Numerical simulation study investigating the effectiveness of ventilation design for hospital wards in terms of virus removal capacity | Low - the estimated effect may be substantially different to the true effect | | | x | | | | | | | |
| Yu, I. T. S., Wong, T. W., Chiu, Y. L., Lee, N., & Li, Y. (2005). Temporal-Spatial Analysis of Severe Acute Respiratory Syndrome among Hospital Inpatients. Clinical Infectious Diseases, 40(9), 91237–91243. Retrieved from https://doi.org/10.1086/428735 | Epidemiological and fluid mechanics study - the temporal and spatial spread of SARS within a hospital ward, Hong Kong, 2003, was compared with computational fluid mechanics modelling of airborne virus concentrations. | High - very confident that the estimated effect is close to the true effect | x | | | | | | | | | |
| Yu, I. T., Li, Y., Wong, T. W., Tam, W., Chan, A. T., Lee, J. H., . . . Ho, T. (2004). Evidence of airborne | Epidemiological and fluid dynamics study investigating correlation between the | Moderate - the estimated effect | x | | | x | | | | | | |

| Reference | Study description | Study quality | 1 – Aerosol transmission | 2 - Faecal-oral | 3 - Ventilation systems | 4 - Plumbing systems | 5 - Fomite transmission | 6 - Residential settings | 7 - Workplaces | 8 - Other indoor settings | 9 - Activities | 10 – Physical distancing |
|---|---|---|---|---|---|---|---|---|---|---|---|---|
| transmission of the severe acute respiratory syndrome virus. N Engl J Med, 350(17), 1731-1739. doi:10.1056/NEJMoa032867 | spatial/temporal distrubution of SARS cases in the Amoy Gardens apartment complex, Hong Kong, 2003, with three-dimensional spread of a virus-laden aerosol plume modeled by computational fluid dynamics | is likely to be close to the true effect but there is a possibility that it is substantially different | | | | | | | | | | |
| Zayas, G., Chiang, M. C., Wong, E., MacDonald, F., Lange, C. F., Senthilselvan, A., & King, M. (2012). Cough aerosol in healthy participants: fundamental knowledge to optimize droplet-spread infectious respiratory disease management. BMC Pulm Med, 12, 11. doi:10.1186/1471-2466-12-11 | Experimental study which measured the size and number of droplets emitted by human subjects whilst coughing in order to characterize the human cough aerosol pattern | High - very confident that the estimated effect is close to the true effect | | | | | | | | | x | |
| Zhang, J., Wang, S., & Xue, Y. (2020). Fecal specimen diagnosis 2019 novel coronavirus-infected pneumonia. J Med Virol, 92(6), 680-682. doi:10.1002/jmv.25742 | Case series – 14 patients, Jinhua, China | Very low - the estimated effect is very uncertain | | x | | | | | | | | |
| Zhou, J., Otter, J. A., Price, J. R., Cimpeanu, C., Garcia, D. M., Kinross, J., . . . Barclay, W. S. (2020). Investigating SARS-CoV-2 surface and air contamination in an acute healthcare setting during the peak of the COVID-19 pandemic in London. Clin Infect Dis. doi:10.1093/cid/ciaa905 | Hospital-based cross-sectional study collecting surface and air samples to test for presence of SARS-CoV-2 RNA, using real-time PCR methods for detection of SARS-CoV-2, London, UK. Study also attempted to culture live virus from environmental samples. | Very low - the estimated effect is very uncertain | x | | | | x | | | | | |
